# Silencing hepatic MCJ attenuates non-alcoholic fatty liver disease (NAFLD) by increasing mitochondrial fatty acid oxidation

**DOI:** 10.1038/s41467-020-16991-2

**Published:** 2020-07-03

**Authors:** Lucía Barbier-Torres, Karen A. Fortner, Paula Iruzubieta, Teresa C. Delgado, Emily Giddings, Youdinghuan Chen, Devin Champagne, David Fernández-Ramos, Daniela Mestre, Beatriz Gomez-Santos, Marta Varela-Rey, Virginia Gutiérrez de Juan, Pablo Fernández-Tussy, Imanol Zubiete-Franco, Carmelo García-Monzón, Águeda González-Rodríguez, Dhaval Oza, Felipe Valença-Pereira, Qian Fang, Javier Crespo, Patricia Aspichueta, Frederic Tremblay, Brock C. Christensen, Juan Anguita, María Luz Martínez-Chantar, Mercedes Rincón

**Affiliations:** 1 CIC bioGUNE, Centro de Investigación Biomédica en Red de Enfermedades Hepáticas and Digestivas (CIBERehd). Bizkaia Science and Technology Park, Derio, Bizkaia Spain; 2 Department of Medicine, Immunobiology Division, University of Vermont, Burlington, VT 05405 USA; 3 Department of Gastroenterology and Hepatology, Marqués de Valdecilla University Hospital, Research Institute Marqués de Valdecilla (IDIVAL), Santander, Spain; 4 Departments of Epidemiology, Pharmacology and Toxicology, and Community and Family Medicine, Geisel School of Medicine at Dartmouth, Lebanon, NH USA; 5 Department of Physiology, Faculty of Medicine and Nursing, University of the Basque Country UPB/EHU. Leioa, Biocruces Health Research Institute, Barakaldo, Spain; 6 Liver Research Unit, Santa Cristina University Hospital, Instituto de Investigación Sanitaria Princesa, CIBERehd, Madrid, Spain; 7 Alnylam Pharmaceuticals, Cambridge, MA USA; 8 Department of Immunology and Microbiology, University of Colorado Denver, Aurora, CO USA; 9 CIC bioGUNE, Inflammation and Macrophage Plasticity laboratory, Bizkaia Science and Technology Park. Derio, Bizkaia, Spain; and Ikerbasque, Basque Foundation for Science, Bilbao, Spain

**Keywords:** siRNAs, Non-alcoholic fatty liver disease

## Abstract

Nonalcoholic fatty liver disease (NAFLD) is considered the next major health epidemic with an estimated 25% worldwide prevalence. No drugs have yet been approved and NAFLD remains a major unmet need. Here, we identify MCJ (Methylation-Controlled J protein) as a target for non-alcoholic steatohepatitis (NASH), an advanced phase of NAFLD. MCJ is an endogenous negative regulator of the respiratory chain Complex I that acts to restrain mitochondrial respiration. We show that therapeutic targeting of MCJ in the liver with nanoparticle- and GalNAc-formulated siRNA efficiently reduces liver lipid accumulation and fibrosis in multiple NASH mouse models. Decreasing MCJ expression enhances the capacity of hepatocytes to mediate β-oxidation of fatty acids and minimizes lipid accumulation, which results in reduced hepatocyte damage and fibrosis. Moreover, MCJ levels in the liver of NAFLD patients are elevated relative to healthy subjects. Thus, inhibition of MCJ emerges as an alternative approach to treat NAFLD.

## Abstract (web summary)

Non-alcoholic fatty liver (NAFLD) disease causes degeneration of the liver, affects about 25% of people globally, and has no approved treatment. Here, the authors show that the therapeutic siRNA-driven silencing of MCJ in the liver is an effective and safe treatment for NAFLD in multiple mouse models.

## Introduction

Nonalcoholic fatty liver disease (NAFLD) is considered a major health epidemic^1,2^. The highest prevalence of NAFLD is found in populations with obesity or type 2 diabetes, but NAFLD is also frequently found in otherwise healthy people without obvious risk factors^1,2^. NAFLD includes a wide spectrum of different pathological conditions^3,4^. While simple symptomatic steatosis (accumulation of lipids in hepatocytes) is the earliest stage of NAFLD, it can progress to non-alcoholic steatohepatitis (NASH), characterized by the presence of fibrosis in the liver^3,4^. NASH is currently the most common cause of chronic liver disease. Patients with NASH have increased liver-mediated mortality and an increased risk of developing cardiovascular diseases. A fraction of NASH patients develops cirrhosis due to progressive liver fibrosis.

NAFLD is initiated by the excessive accumulation of lipids in the liver due to an increased delivery of fatty acids from adipose tissue accompanied by an imbalance between lipid degradation and de novo lipid synthesis^1,2^. The accumulation of lipids within hepatocytes triggers a chronic inflammatory response that involves other hepatic cell types, such as Kupffer and stellate cells, and can lead to liver fibrosis (NASH). Fatty acids are catabolized in the liver by β-oxidation within mitochondria, with the resulting reducing products (NADH and FADH_2_) being fed into the mitochondrial electron transport chain (ETC). Lipid catabolism in the liver is therefore highly dependent on mitochondrial metabolism. Increasing mitochondrial respiratory activity in the liver could enhance degradation of fatty acids, thereby preventing their accumulation and liver disease progression. However, this pathway has not been extensively considered therapeutically for the treatment of NAFLD because of the potential increase in the generation of reactive oxygen species (ROS) as a result of enhanced β-oxidation.

MCJ is a transmembrane protein in the inner mitochondrial membrane (encoded by the *Dnajc15* nuclear gene) that acts as an endogenous negative regulator of Complex I and restrains mitochondrial respiration^5^. Loss of MCJ leads to increased Complex I activity, mitochondrial membrane potential, and mitochondrial respiration^5–7^. However, loss of MCJ does not increase the production of ROS because it also promotes the formation of respiratory supercomplexes that are commonly formed by Complex I, III, and IV of the ETC^5–7^. The major function of supercomplexes is to facilitate the transport of electrons between Complex I to III and III to IV by bringing them into proximity, and thereby minimize electron leak^8,9^. MCJ is expressed predominantly in highly metabolic tissues including the liver, heart, and kidney^5^. Under normal physiological conditions MCJ is dispensable as no obvious abnormalities can be found in MCJ-deficient mice^5^. Disrupting MCJ expression and/or function in the liver could, therefore, be a safe strategy to minimize lipid accumulation and the development of fibrosis, and thereby treat NASH. In this study we show that MCJ-deficient mice are resistant to the development of fatty liver and NASH. Importantly, using siRNA as a therapeutic approach we show that treatment with different formulations of siMCJ after the onset of the disease reduces liver steatosis and fibrosis in multiple mouse models. These results, together with the increased levels of MCJ in liver of NAFLD patients we report here, suggest that MCJ is emerging as an alternative target for treatment of NASH.

## Results

### Reduced NAFLD liver steatosis and fibrosis in MCJ deficient mice

**Fig. 1:**
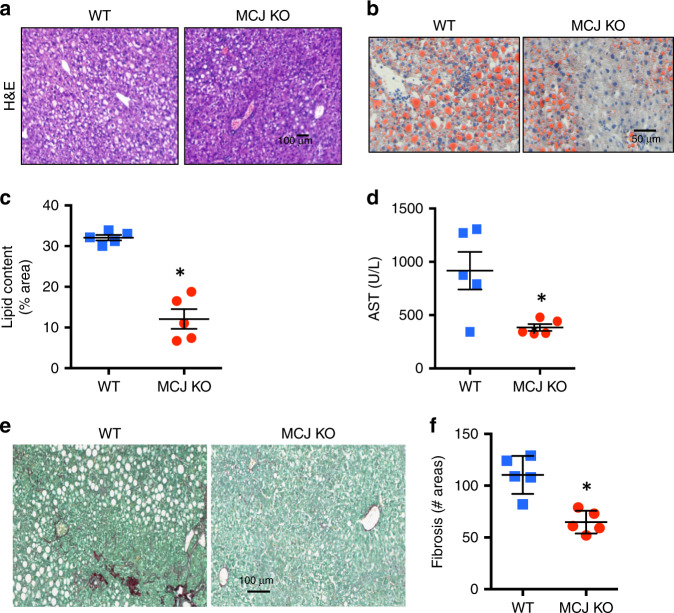
MCJ deficient mice are resistant to develop liver steatosis and fibrosis in the MCD diet model. (**a**–**d**) WT (*n* = 5) and MCJ KO (*n* = 5) mice were placed on MCD (methionine- and choline-deficient) diet for 2 weeks prior to liver harvest. **a** Representative liver H&E images from WT and MCJ KO mice. **b** Representative images and (**c**) quantitation of liver lipid content determined by Sudan III staining and shown as percentage of the total histological area. (**d**) Serum AST levels. **e, f** WT (*n* = 5) and MCJ KO (*n* = 5) mice were placed on MCD diet for 3 weeks prior to liver harvest. **e** Representative images and (**f**) quantification of liver fibrosis determined by Picro Sirius Red staining and shown as the number of fibrotic areas per histological section. *denotes *p* < 0.05 as determined by Student´s *t* test. Error bars show standard error (SE) in (**c**) and (**d**), and standard deviation (SD) in (**f**). Source data are provided as a Source Data file.

MCJ is a negative regulator of respiratory Complex I and mitochondrial respiration^5–7^. MCJ is expressed in the liver^5–7^, markedly at higher levels in hepatocytes than in other liver resident cells such as Kupffer cells and stellate cells (Supplementary Fig. 1). We therefore investigated the role of MCJ in fatty liver disease using MCJ-deficient (MCJ KO) mice and the methionine- and choline-deficient (MCD) diet mouse model of NASH where lipid accumulation in the liver causes steatosis followed by fibrosis. WT and MCJ KO mice were maintained for 2 weeks on the MCD diet. Histological analysis of the liver showed profound steatosis in WT mice, but minimal steatosis was found in MCJ KO mice (Fig. 1a). The analysis of lipid accumulation showed reduced accumulation of lipids in the livers of MCJ KO mice relative to WT mice (Fig. 1b, c). Protection from lipid-mediated liver damage in MCJ KO mice was further demonstrated by the lower levels of serum aspartate aminotransferase (Fig. 1d). To examine whether loss of MCJ could also impact liver fibrosis, WT and MCJ KO mice were maintained on the MCD diet for 3 weeks. Liver fibrosis was determined by Picro Sirius Red staining of liver sections. Similar to liver steatosis, MCJ KO mice developed less fibrosis in the liver relative to WT mice (Fig. 1e, f). Thus, systemic loss of MCJ protects mice from developing NASH.

### Decreased MCJ gene methylation and increased expression in human NAFLD

**Fig. 2:**
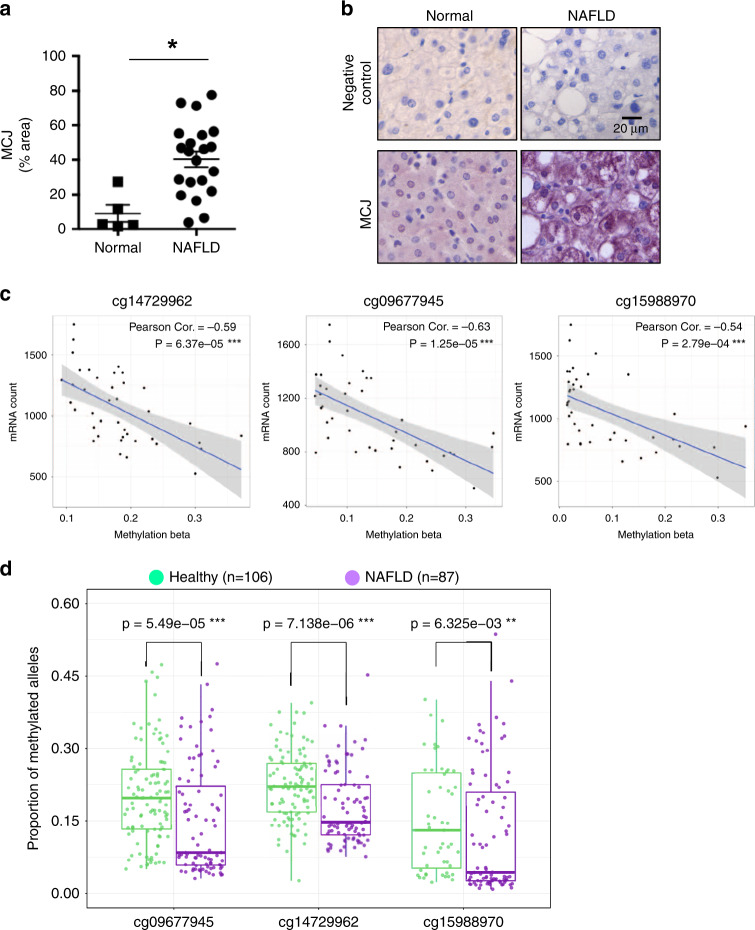
Increased MCJ expression in the liver correlates with the development of NAFLD in humans. **a** Quantification and (**b**) representative images of liver MCJ expression determined by immunohistochemistry in human samples from healthy control subjects (Normal) (*n* = 5) and patients with non-alcoholic fatty liver disease (NAFLD) (*n* = 21). *denotes *p* < 0.05 as determined by Student’s *t* test. Error bars show standard error (SE). **c** Linear regression analysis for the methylation beta scores and DNAJC15 mRNA levels of the 3 CpG sites identified in Supplementary Fig 2a, b. Results show a strong negative association (all Pearson correlation coefficients <−0.50 and linear regression *P* < 0.001). **d** Wilcoxon’s rank-sum test comparing methylation levels in NAFLD patients and healthy human liver at three gene-expression-related CpG within the *DnaJC15* promoter, based on six independent GEO data sets. **P < 0.01, ***P < 0.001. Error bars show standard error (SE) in (**a**). Source data are provided as a Source Data file.

*DNAJC15* has been recently identified as one of the genes differentially expressed in humans in association with metabolic disorders^10,11^. No studies to date have investigated MCJ/*DNAJC15* in NAFLD patients. We therefore examined MCJ protein levels by immunohistochemistry using a specific anti-MCJ monoclonal antibody^12^ in liver biopsies from a cohort of patients with biopsy-proven NAFLD as well as from a cohort of healthy subjects. Increased MCJ levels were found in livers from NAFLD patients compared with healthy controls (Fig. 2a, b), supporting a role of MCJ in NAFLD.

The expression of MCJ is known to be downregulated by gene promoter DNA methylation in different types of cancer cells^13–15^. However, it is unclear whether regulation of *DNAJC15* expression by DNA methylation occurs in nontumor liver tissues. We have recently identified three CpG sites (cg14729962, cg09677945, cg15988970) within the proximal promoter of human *DNAJC15* where methylation inversely correlates with expression in human breast cancer^15^. Here, we integrated and analyzed *DNAJC15* DNA methylation data in healthy liver tissues from six independent Gene Expression Omnibus (GEO) data sets and The Cancer Genome Atlas (TCGA), and observed the highest variability in methylation of these three CpG sites relative to the other CpG sites (Supplementary Fig. 2a, b). Consistent with our published results in breast tissues^15^, methylation of the three *DNAJC15* CpG sites was inversely correlated with gene expression in normal TCGA liver tissues, where lower DNA methylation was associated with higher levels of *DNAJC15* transcripts all Pearson Corr. <−0.5 and *P* < 0.001, (Fig. 2c). We then compared the *DNAJC15* methylation profile of these three sites in livers from healthy (*n* = 106) and NAFLD patients (*n* = 87) in the six GEO data sets. These three *DNAJC15* promoter CpGs were hypomethylated in NAFLD patient livers relative to healthy livers at the three CpG sites related to *DNAJC15* expression (all Wilcoxon’s test *P* < 0.01, Fig. 2d). Thus, consistent with the increased levels of MCJ protein expression in liver of NAFLD patients, these results further support the association of liver MCJ levels and human NAFLD.

### LNP-siMCJ treatment reduces liver steatosis and fibrosis in MCD diet models

**Fig. 3:**
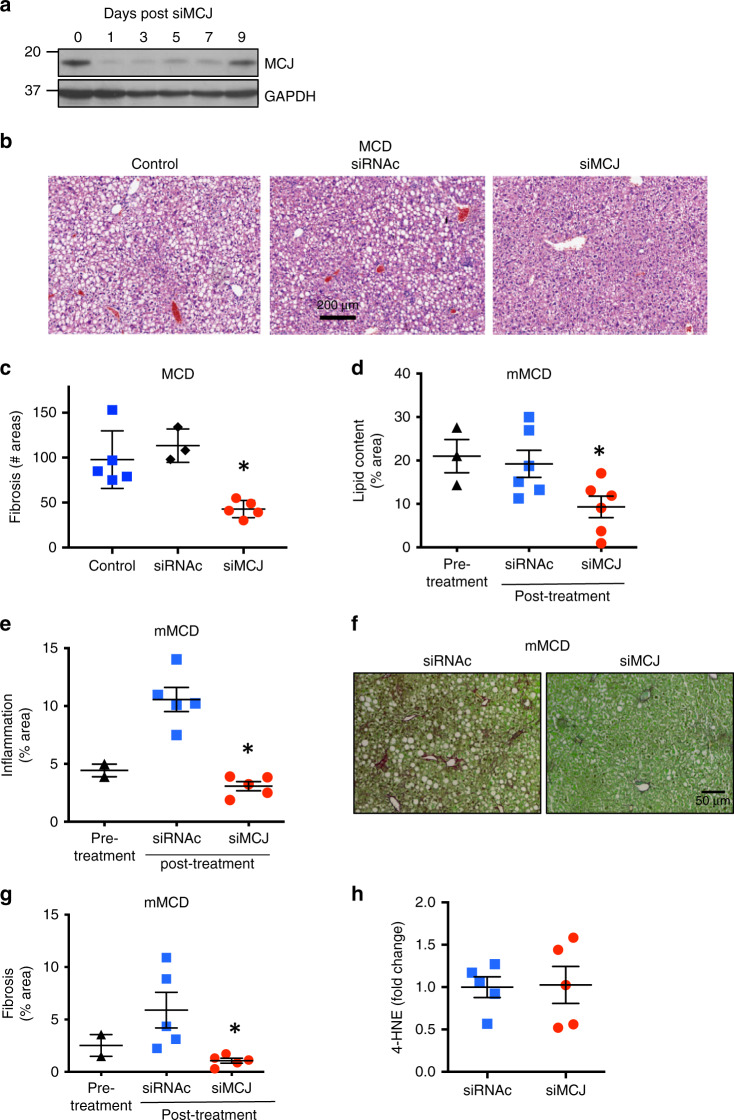
Reducing MCJ levels in the liver by in vivo administration of siMCJ decreases liver steatosis and fibrosis in the MCD diet models. **a** WT mice received a single i.v. dose (1.7 mg/Kg) of siMCJ using Invivofectamine as a delivery system. Livers were harvested at the times indicated and MCJ levels were examined by Western blot analysis. **b** and **c** WT mice were placed on MCD diet and received an i.v. administration of Invivofectamine-formulated siMCJ (*n* = 5), Invivofectamine-formulated nonspecific control siRNA (siRNAc) (*n* = 3), or PBS (*n* = 5) weekly starting at the initiation of the diet. Mice were harvested after 3 weeks on MCD diet (1 week after the last dose of siMCJ). **b** Representative images of liver lipids by H&E staining of liver sections are shown. **c** Number of fibrotic areas per histological section of the liver as determined by Picro Sirius Red staining. **d**–**g** WT mice were placed on mMCD for the duration of the study. After 1 week on mMCD diet (pre-treatment), mice were administered weekly i.v. dose of Invivofectamine-formulated siMCJ (*n* = 6) or siRNAc (*n* = 6) for a total of 3 weeks and harvested 1 week after the last dose (4 weeks total on the diet). **d** Quantification of liver lipid content by Sudan III staining of histological sections. **e** Quantification of liver inflammation by F4/80 immunostaining of histological sections. **f** Representative images and (**g**) quantitation of liver fibrosis by Picro Sirius Red staining. **h** Quantification of 4-HNE staining of liver sections. *p < 0.05 siMCJ-treated compared with PBS control by one-way ANOVA or Student’s *t* test analysis. Error bars show standard error (SE) in (**d**), (**e**), (**g**) and (**h**), and standard deviation (SD) in (**c**). Source data are provided as a Source Data file.

The results above suggested that MCJ could be a potential target for NAFLD. We investigated whether MCJ could be therapeutically targeted in the liver as an approach to treat NASH. siRNA successfully attenuates the expression of liver proteins, and the first siRNA therapies were recently approved by FDA for a rare liver disease^16,17^. We followed the same approach to target MCJ in the liver using a siRNA specific for mouse *Dnajc15* (siMCJ) previously validated as a shRNA plasmid^18^. To determine the efficacy of siMCJ in reducing MCJ expression in vivo, WT mice were i.v. administered a single dose of siMCJ using Invivofectamine (lipid-based) as a delivery system that resembles the lipid nanoparticles (LNP) delivery system used for one of the FDA-approved siRNA-based therapies. Livers were harvested at different time points after siMCJ administration and levels of MCJ were examined by Western blot analysis. As early as 24 h after the administration of siMCJ, endogenous MCJ was almost undetectable in the liver (Fig. 3a). The levels of MCJ remained low for up to 7 days following siMCJ administration (Fig. 3a). No effect on MCJ levels was observed when a control siRNA was administered (Supplementary Fig. 3a). In contrast to MCJ in liver, siMCJ treatment did not obviously affect MCJ levels in the heart or kidneys (Supplementary Fig. 3b), two other tissues where MCJ is highly expressed^5^. Thus, siMCJ administration was highly efficient in reducing the levels of MCJ in the liver. To address potential toxicity of the siMCJ treatment, mice were administered a dose fivefold higher than the experimental dose and markers of toxicity were examined 1 week later. Serum levels of transaminases, albumin (liver dysfunction), and urea (renal toxicity) were not elevated in mice that received high doses of siMCJ compared with control mice (Supplementary Fig. 4a, b, c).

We therefore investigated the effect of siMCJ administration as therapy in mouse models of fatty liver disease. We first tested acute fasting as a model of early phase NAFLD because mice develop liver steatosis with a marked lipid accumulation due to the rapid and overwhelming hydrolysis of lipids in the adipose tissue. We have previously shown that MCJ deficiency prevents acute lipid accumulation in the liver during fasting^5^. WT mice were given a single i.v. dose of siMCJ 18 h prior to food withdrawal, and mice were euthanized after 36 h of fasting. Analysis of MCJ in the liver by Western blot analysis showed a clear reduction (Supplementary Fig. 5a). While control mice showed a massive accumulation of lipids in the liver, minimal lipids could be found in siMCJ-treated mice (Supplementary Fig. 5b, c). Thus, targeting MCJ in the liver by administering siMCJ is an effective way to reduce lipid accumulation.

We then assessed whether the administration of siMCJ could prevent the accumulation of lipids in the liver and development of NASH using the MCD diet model. Based on the pharmacodynamics shown above, a weekly i.v. dose of siMCJ was chosen starting simultaneously with the MCD diet. As controls, mice received either no siRNA (control) or a non-specific siRNA control (siRNAc). Mice were analyzed after 3 weeks on MCD diet (1 week after the last siMCJ treatment). The levels of MCJ remained low in siMCJ treated mice compared with control animals even 1 week after the last treatment (Supplementary Fig. 6a). Relative to the livers of control and control-siRNA treated mice, only minor liver steatosis was present in the livers of siMCJ-treated mice, as determined by H&E staining (Fig. 3b), and lipid staining of liver sections (Supplementary Fig. 6b). Thus, inhibition of MCJ expression in the liver is an efficient approach to prevent progressive lipid accumulation. Analysis of liver fibrosis by Picro Sirius Red staining revealed markedly reduced fibrosis in siMCJ-treated mice (Fig. 3c and Supplementary Fig. 6c). Thus, administration of siMCJ simultaneously with MCD diet prevents the development of NASH in mice.

To examine the therapeutic value of siMCJ after the onset of the disease, we used a modified MCD diet (mMCD diet) that is fully deficient in choline but contains a small amount (0.1%) of methionine. Mice can be maintained on this diet for 4 weeks or longer with no substantial weight loss^19^. siMCJ treatment was initiated one week after starting the diet, when the mice already displayed very high liver lipid content (Fig. 3d), mild inflammation (Fig. 3e), and signs of fibrosis (Fig. 3f, g). The mice were maintained on mMCD diet for an additional 3 weeks with a weekly dose of siMCJ and euthanized 1 week after the last dose (4 weeks on diet total). The efficient suppression of MCJ expression in the liver was confirmed by Western blot analysis (Supplementary Fig. 7a). Analysis of H&E staining of liver sections showed minimal levels of steatosis in siMCJ-treated mice relative to controls (Supplementary Fig. 7b). In addition, histochemical staining analysis revealed low lipid content in the livers of siMCJ-treated mice compared with nontreated mice and mice fed mMCD diet for only 1 week (Fig. 3d and Supplementary Fig. 7c). Thus, treatment with siMCJ after the onset of liver steatosis and development of fatty liver reduces the lipid content of the liver. While enhanced β-oxidation and mitochondrial ETC activity may lead to increase ROS production due to electron leak, we have shown that loss of MCJ increases Complex I activity and mitochondrial respiration without increasing ROS because it promotes the formation of supercomplexes that reduce electron leak^5–8,20^. Under physiological conditions, MCJ deficient hepatocytes have increased mitochondrial respiration but have no elevated levels of ROS despite having an increased Complex I activity^6^. We evaluated hepatic ROS in the mice on mMCD diet by immunohistochemical staining for 4-hydoxynonenal (4-HNE), an aldehyde product of lipid peroxidation used as a sensitive marker of oxidative damage and lipid peroxidation^21,22^. The levels of 4-HNE were not increased in siMCJ-treated mice groups (Fig. 3h) demonstrating that hepatic ROS production is not increased by MCJ silencing.

Correlating with the lower lipid accumulation, livers from siMCJ-treated mice also had less hepatocyte damage as indicated by reduced serum ALT levels (Supplementary Fig. 7d) and diminished liver inflammation determined by the reduced presence of F4/80-myeloid cells (Fig. 3e and Supplementary Fig. 7e). Importantly, analysis of liver fibrosis, as a hallmark of NASH, revealed markedly lower fibrosis in livers from siMCJ-treated mice (Fig. 3f, g). Thus, treatment with siMCJ after the onset of the disease prevented the development of liver fibrosis by reducing lipid content in the liver.

### Efficacy of LNP-siMCJ treatment in a metabolic disorder model of NASH

**Fig. 4:**
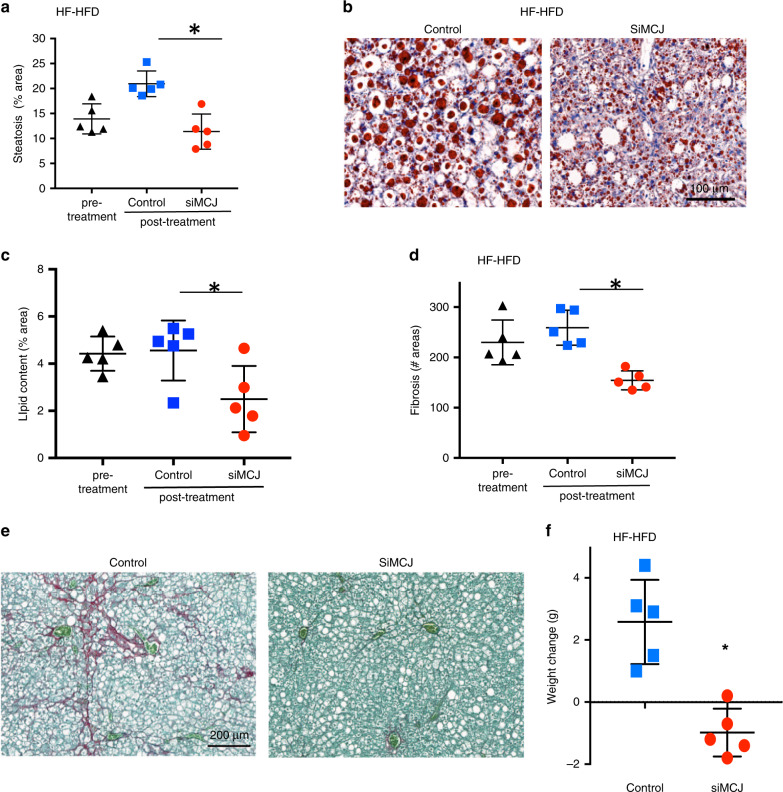
siMCJ treatment decreases liver steatosis, fibrosis and weight gain in a NASH model with metabolic disorders. Mice were placed on a high-fat, high-fructose diet (HF-HFD) for the duration of the study. Treatment with Invivofectamine-formulated siMCJ (*n* = 5) or PBS (*n* = 5) started after 4 months on HF-HFD (pre-treatment) and was provided weekly for a total of 5 weeks. Tissues were harvest 1 week after the last treatment. **a** Quantification of liver steatosis in H&E staining histological sections. **b** Representative images of liver lipids by Oil Red O staining. **c** Quantification of lipids from Oil Red O stained-liver sections. **d** Number of fibrotic areas per histological section of the liver as determined by Picro Sirius Red staining. **e** Representative images of Picro Sirius Red-stained liver sections. **f** Weight change after 6 weeks of treatment. *p < 0.05 siMCJ-treated compared with PBS control by one-way ANOVA or Student´s *t* test analysis. Error bars show standard deviation (SD) in all panels. Source data are provided as a Source Data file.

Considering that NASH is frequently associated with metabolic disorders such as obesity and diabetes, we investigated whether siMCJ treatment could be effective in the high fat-high fructose diet (HF-HFD)^23^ model (resembling the Western diet) where mice develop NASH. Mice were maintained on this diet for 4 months prior to the initiation of the treatment. At this time, the mice had already developed liver steatosis (Fig. 4a, b, c) and fibrosis (Fig. 4d, e) and had an average weight of 50 g (Fig. 4f). The mice were then treated with one dose of siMCJ per week for a total of 5 weeks (5 doses total) and euthanized 1 week after the last dose (5.5 months on the diet). The levels of MCJ in the liver of siMCJ-treated mice remained low 1 week after the last dose (Supplementary Fig. 8a). Liver steatosis was markedly reduced in siMCJ-treated mice, as determined by H&E staining (Fig. 4a and Supplementary Fig. 8b) as well as by lipid staining in histological liver sections (Fig. 4b, c). In addition, liver fibrosis was also reduced in siMCJ-treated mice as determined by the number of fibrotic areas in the liver sections (Fig. 4d) as well as the relative collagen area (Fig. 4e and Supplementary Fig. 8c). Thus, treatment with siMCJ has efficacy in improving liver steatosis and fibrosis in NASH under metabolic disorder conditions.

Recent studies have suggested that the capacity of the liver to metabolize fat could have an impact in the overall metabolism and weight gain^24^. Blood glucose and serum insulin levels were not affected by the treatment with siMCJ (Supplementary Fig. 8d, e). However, while the weight of nontreated mice continued to increase over the 6 weeks following the beginning of the treatment, the weight of siMCJ-treated mice decreased (Fig. 4f). To further support a potential benefit of disrupting MCJ in the liver on metabolic disorders, we tested the effect of siMCJ in weight gain in LepR^db^ mice, which show a continued increased weight due to a genetic mutation in the leptin receptor leading to a deficiency in leptin-mediated signaling^25^. Treatment started at 5 weeks of age and continued for 4 weeks. siMCJ also reduced weight gain in this genetic mouse model (Supplementary Fig. 9). Thus, reducing MCJ expression in the liver through the treatment with siMCJ also contributes to minimize weight gain.

Insulin resistance and diabetes are also frequently found in obese patients with NAFLD, although it is unclear whether this is dependent on liver function or constitutes a comorbidity factor^1,2^. We therefore tested whether silencing MCJ in the liver and increasing hepatic mitochondrial function could affect insulin resistance by treating mice on high fat diet for 13 weeks (known to exhibit insulin resistance) with siMCJ. After 2 weeks of treatment with siMCJ, an insulin tolerance test was performed by determining blood glucose levels. siMCJ treatment increased insulin response compared with control-treated mice (Supplementary Fig. 10). These results show the efficacy of siMCJ as a treatment for NASH in the context of obesity while managing metabolic disorders.

To further demonstrate the effect of siMCJ treatment on liver fibrosis we also examined its effect on aggressive liver fibrosis caused by the administration of carbon tetrachloride (CCl_4_), the most frequently used model to investigate liver fibrosis^26^. After 2 weeks of periodical i.p. injections of CCl_4_, mice were treated weekly with siMCJ or vehicle control for 3 additional weeks (total of 5 weeks with CCl_4_ administration). As reported, the levels of serum ALT were only slightly elevated, and there was no difference in the two groups (Supplementary Fig. 11a). Liver fibrosis was evaluated by Picro Sirius Red staining of liver sections. Unlike the diet models of NASH where fibrosis develops mostly in perivenular area, mice treated with CCl_4_ develop a pattern of fibrotic bridging between central and portal areas that resembles that observed in advanced stage human NASH. As previously described, after 2 weeks of CCl_4_ administration, fibrosis was present but minimal signs of bridging could be found (Supplementary Fig. 11b, c). After 5 weeks of CCl_4_ administrations, marked bridging was present in mice from the control group (Supplementary Fig. 11b, c). In contrast, while fibrosis was present, fibrotic bridging was clearly reduced in the siMCJ-treated mice (Supplementary Fig. 11b, c). Thus, treatment with siMCJ after fibrosis is established reduces the progression to more advance and detrimental stages of liver fibrosis.

### siMCJ treatment lowers hepatocyte lipid levels by enhancing β-oxidation

**Fig. 5:**
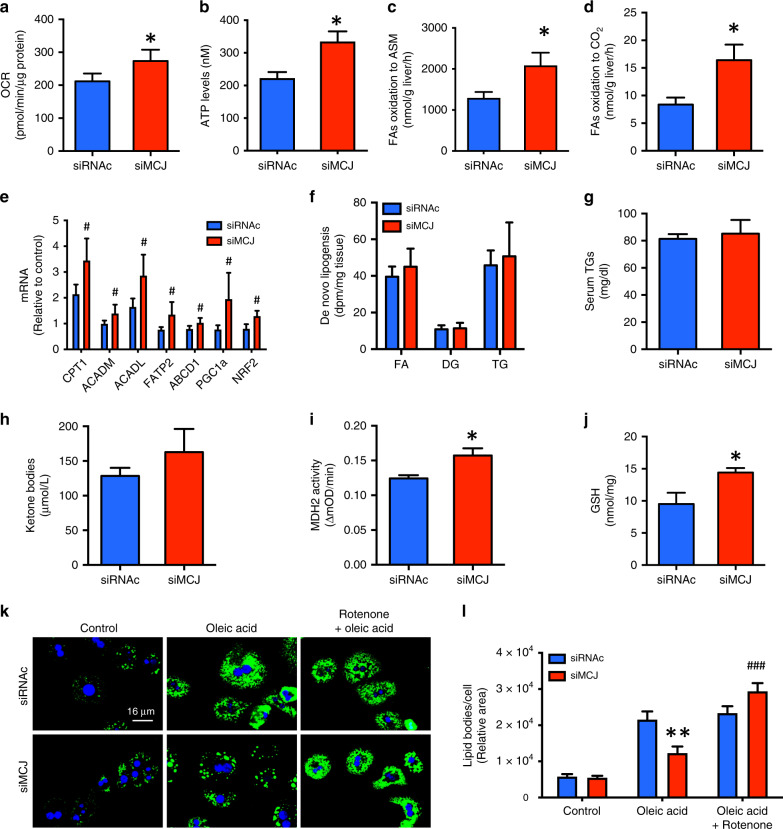
MCJ silencing enhances β-oxidation and prevents lipid accumulation in hepatocytes in vitro and in vivo. **a**–**j** Mice were placed on mMCD diet 1 week prior to the initiation of the treatment and maintained on the diet for the duration of the study. Treatment with Invivofectamine-formulated siMCJ (*n* = 5) or siRNAc (*n* = 5) was given weekly for 3 weeks. Tissues were harvested 1 week after the last dose. **a** Oxygen consumption rate (OCR) using the Seahorse analyzer. **b** ATP levels in isolated mitochondria. **c** Rate of fatty acid oxidation determined by the production of acid soluble metabolites (ASM). **d** Rate of fatty acid oxidation determined by the production of CO_2_. **e** mRNA expression of fatty acid oxidation genes in siMCJ-treated liver relative to control liver, as determined by real time RT-PCR. **f** De novo lipogenesis of fatty acids (FA), diglycerides (DG), and triglycerides (TG) in the liver. **g** Triglycerides levels and (**h**) ketone bodies levels in serum. **i** Activity of malate dehydrogenase (MDH2) in liver extracts. **j** Glutathione (GSH) levels in liver. **k**-**l** Primary hepatocytes from WT mice were transfected with siRNAc or siMCJ and were incubated with steatosis-inducing doses of oleic acid alone for 24 h or in combination with rotenone for the last 6 h. **k** Representative images of lipid content and (**l**) quantification evaluated by Bodipy staining. *denotes *p* < 0.05, as determined by Student’s *t* test analysis. Error bars show standard error (SE) in all the panels except panels (**e**) and (**f**) where error bars show standard deviation (SD). Source data are provided as a Source Data file.

Although the initial causes of NAFLD remain undefined, the pathological accumulation of lipids in the liver seems to result from an imbalance between acquisition of lipids by de novo lipogenesis or uptake, and catabolism of the lipids through β-oxidation of fatty acids in mitochondria. The byproducts of β-oxidation, NADH and FADH_2_, directly feed into the ETC to be oxidized by Complex I and Complex II, respectively. Since MCJ is a negative regulator of Complex I, removing this restraint on Complex I activity could enhance the oxidation of NADH and thereby enhance fatty acid β-oxidation. We first tested whether silencing MCJ could increase mitochondrial respiration in the fatty liver using the mMCD diet model. Mice were placed on mMCD diet for 4 weeks and treatment with siMCJ started 1 week after the initiation of the diet. Mitochondria were then isolated from the livers of mMCD diet-fed mice and respiration in freshly isolated mitochondria was examined using the Seahorse MitoStress assay. The oxygen consumption rate (OCR) was elevated in mitochondria from siMCJ-treated mice compared with nontreated mice (Fig. 5a). Accordingly, mitochondrial ATP levels were also increased in siMCJ-treated mice (Fig. 5b). Thus, reducing MCJ in vivo by treatment with siMCJ in NAFLD enhances mitochondrial respiration. We then tested whether siMCJ treatment could also indirectly enhance fatty acid β-oxidation in NAFLD. Using fresh liver sections from control and siMCJ-treated mice on mMCD diet, analysis of fatty acid β-oxidation by acid soluble metabolites (ASM) (Fig. 5c) and CO_2_ release (Fig. 5d) revealed a significant increase in β-oxidation in the livers of siMCJ-treated mice. Consistent with these results, the expression of genes involved in mitochondrial β-oxidation (*Cpt1, Acadm, Acadl, Fatp2, Abcd1, Pgc1α*, and *Nrf2*) was also elevated in the livers from siMCJ-treated mice (Fig. 5e). Thus, treatment of NAFLD with siMCJ enhances fatty acid β-oxidation in vivo. In contrast, analysis of de novo lipogenesis using fresh liver sections showed comparable levels of de novo synthesis free fatty acids, diglycerides, and triglycerides in the liver between control and siMCJ-treated mice (Fig. 5f). Moreover, the levels of TG in the serum were low in both control and siMCJ-treated mice on mMCD (Fig. 5g), indicating that the export of TG is not affected by siMCJ treatment. Thus, siMCJ-treatment reduces liver lipid accumulation in NAFLD by enhancing mitochondrial β-oxidation and catabolism of the lipids.

Although excessive β-oxidation could lead to increased production of ketone bodies, serum ketone bodies were not elevated in siMCJ-treated mice relative to control mice on mMCD diet (Fig. 5h). Since we have previously shown that the loss of MCJ in deficient mice enhances TCA cycle activity as a result of increased mitochondrial ETC activity^5,7^, the acetyl-CoA generated during β-oxidation could be redirected to the TCA cycle instead of the production of ketone bodies. Accordingly, analysis of the TCA cycle enzyme malate dehydrogenase (MDH2) activity showed that MDH2 activity was significantly higher in livers from siMCJ-treated mice compared with control mice on mMCD diet (Fig. 5i). We also examined ROS production using the glutathione (GSH) levels as a surrogate marker (low GSH indicative of elevated oxidative stress since its antioxidant activity is used to neutralize ROS). Interestingly, despite increased β-oxidation and mitochondrial respiration, the levels of GSH in livers from siMCJ-treated mice remained higher than in control mice (Fig. 5j). Thus, targeting MCJ in the liver is a alternative safe approach to treat NAFLD as it prevents lipid accumulation by boosting mitochondrial respiration and β-oxidation without causing oxidative stress in the liver or ketosis.

To further demonstrate that the enhanced lipid catabolism in the liver resulting from siMCJ-treatment is due to increased Complex I activity in hepatocytes, we inhibited MCJ expression in isolated primary hepatocytes from WT mice in vitro by transfecting with siMCJ. siRNAc- and siMCJ-transfected hepatocytes were then incubated with a steatotic-dose of oleic acid in the absence or presence of rotenone, a pharmacological inhibitor of Complex I. Lipid content was quantified using BODIPY. Consistent with the in vivo studies, attenuation of MCJ expression prevented accumulation of lipids in hepatocytes (Fig. 5k, l). However, inhibition of Complex I restored lipid accumulation in the absence of MCJ (Fig. 5k, l). Thus, increasing ETC activity through attenuation of MCJ expression is a unique approach to accelerate lipid catabolism and prevent lipid accumulation in the liver.

### Therapeutic GalNAc-siMCJ reduces NASH liver steatosis and fibrosis

**Fig. 6:**
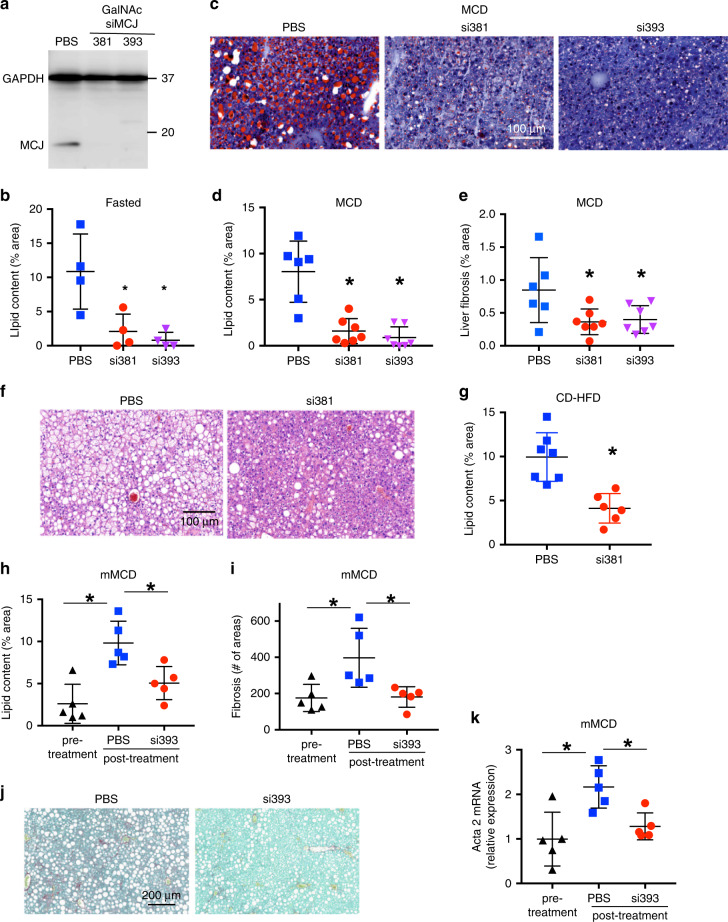
Treatment with GalNAc-siMCJ attenuates fatty liver disease. **a** Mice received a single s.c. dose of GalNAc-si381 (10 mg/Kg), GalNAc-si393, or PBS. MCJ expression in the liver was analyzed 5 days later by Western blot analysis. **b** Mice (*n* = 4) received a single dose of GalNAc-si381 or GalNAc-si393 (10 mg/Kg), or PBS. After 5 days they were fasted for 36 h. Liver lipids were quantified by Oil Red O staining. **c**–**e** Mice were administered with a s.c. dose of GalNAc-si381 or GalNAc-si393 (*n* = 7), or PBS (*n* = 6) at the time they started on the MCD diet. A second dose was administered 2 weeks later. Mice were harvested after 1 additional week. **c** Representative images of liver lipid accumulation by Oil Red O staining. **d** Quantitation of liver lipids. **e** Quantitation of liver fibrosis by Picro Sirius Red staining. **f** and **g** Mice (*n* = 7) were fed choline-deficient high-fat diet (CD-HFD) for 4 months prior to the treatment with a s.c. dose of GalNAc si381 or PBS every 2 weeks for 2 more months on the diet (4 doses total). Tissues were harvested 2-weeks after the last dose. (**f**) Representative images of H&E stained-liver sections. (**g**) Quantification of liver lipids by Oil Red O staining. Percentage of total histological area is shown. **h**–**k** After 2 weeks on mMCD diet (pre-treatment), mice (*n* = 5) received s.c. administration of GalNAc-si393 or PBS every 2 weeks for 4 additional weeks (2 doses total). Tissues were harvested 2 weeks after the last dose. **h** Quantitation of liver lipid by Oil Red O staining of liver sections. **i** Quantitation of liver fibrosis by Picro Sirius Red staining (number of fibrotic areas per section). **j** Representative liver images of Picro Sirius Red staining. **k** Relative expression of smooth muscle actin (Acta 2) mRNA levels by real-time RT-PCR and β2-microglobulin as house-keeping gene. *denotes *p* < 0.05, as determined by one-way ANOVA or *t* test. Error bars show standard deviation (SD) in all panels. Source data are provided as a Source Data file.

Considering that hepatocytes are the preferential target for LNP^27^ and that MCJ is more abundantly expressed in liver hepatocytes relative to other liver resident cells^6^ (Supplementary Fig. 1), silencing MCJ in hepatocytes is most likely the main mechanism for the siMCJ/Invivofectamine effect in vivo. Nevertheless, to further verify the beneficial therapeutic effect of silencing MCJ in hepatocytes, as an alternative to LNP, we examined the use of N-Acetylgalactosamine (GalNAc) as a delivery system for siRNA specifically to hepatocytes^17,28,29^. GalNAc is the preferred method of siRNA delivery for treatment of liver diseases that require extended periods of treatment and it has been recently approved by the FDA as a delivery system for siRNA in a liver rare disease. The major advantages of GalNAc as a siRNA vehicle relative to LNP are its reduced immunogenicity, its selectivity to hepatocytes (due to the expression of GalNAc receptors), its s.c. administration (instead of i.v.), and its prolonged effect^17,28,29^. We therefore investigated the therapeutic effect of GalNAc-siMCJ. Several stabilized siRNA sequences specific for MCJ were conjugated to GalNAc, tested for their capacity to silence MCJ gene expression in vitro and in vivo and two GalNAc-siRNA sequences for MCJ (si381 and si393) were selected for their superior efficacy in silencing MCJ gene expression (Supplementary Fig. 12a). The efficacy of si381 and si393 to lower MCJ protein levels in the liver was further confirmed by Western blot analysis (Fig. 6a). As expected, due to the slower release of the GalNAc formulation, the kinetics of loss were delayed relative to Invivofectamine, but by 3 days after s.c. administration of si381, MCJ was undetectable in the liver (Supplementary Fig. 12b). We first tested whether silencing MCJ in hepatocytes with si381 and si393 had similar effect in reducing lipid accumulation in the liver using the fasting model. After 36 h of fasting, no MCJ could be detected in the livers from mice treated with si381 or si393 (Supplementary Fig. 13a). There was almost no detectable liver steatosis in mice treated with si381 or si393 relative to mice treated with PBS, either by histological analysis (Supplementary Fig. 13b, c) or by lipid staining in liver sections (Fig. 6b and Supplementary Fig. 13d).

We also examined the efficacy of GalNAc-siMCJ in the MCD diet model. Administration of si381 and si393 was initiated at the time the mice were placed on the diet. A second dose was provided 2 weeks later, and mice were harvested 1 week later. The levels of MCJ in the liver remained markedly reduced in the treated mice a week after the last treatment (Supplementary Fig. 14a). Minimal lipid accumulation in the liver could be detected in mice treated with si381 or si393, relative to control mice (Fig. 6c, d). Importantly, treatment with si381 and si393 suppressed the development of fibrosis (Fig. 6e and Supplementary Fig. 14b).

To test the effect of a prolonged treatment with GalNAc-siMCJ we used a choline-deficient high-fat diet (CD-HFD) model of NAFLD. Mice were maintained in the diet for 4 months prior to the initiation of the treatment, and were treated with si381 for 2 months with a dose every 2 weeks (total of 4 doses). The levels of MCJ in the liver remained very low even 2 weeks after the last dose of si381 (Supplementary Fig. 15a). Liver steatosis as determined by H&E histological analysis was markedly reduced in si381-treated mice relative to the control group (Fig. 6f and Supplementary Fig. 15b). In addition, while extensive accumulation of fat in the liver is achieved in this model, liver lipid content was also highly reduced in mice treated with si381 (Fig. 4g and Supplementary Fig. 15c).

To further test the efficacy of GalNAc-formulated MCJ siRNA on the treatment of fibrosis in NASH after the onset of the disease, we used the mMCD diet model. After 2 weeks on the mMCD diet, mice had already developed significant steatosis as determined by lipid accumulation in the liver (Fig. 6h and Supplementary Fig. 16a) and histological analysis (Supplementary Fig. 16b), as well as liver fibrosis (Fig. 4i). Mice were then treated with si393 or PBS every 2 weeks for 4 additional weeks (2 doses). Lipid accumulation (Fig. 6h and Supplementary Fig. 16a) and overall histological liver steatosis (Supplementary Fig. 16b) were markedly reduced in si393-treated mice compared with the control group. In addition, while liver lipid content increased in PBS-treated mice relative to the initiation of the treatment, no difference was detected in si393-treated mice (Fig. 6h). Importantly, fibrosis analysis also revealed substantially lower levels of liver fibrosis in si393-treated mice relative to the PBS-treated group, as determined by Picro Sirius Red staining (Fig. 6i, j), as well the levels of smooth muscle actin in liver (Fig. 6k). Similar to lipid content, liver fibrosis in si393-treated mice was no different from the levels of fibrosis at the initiation of the treatment (Fig. 6i, k), indicating that treatment with GalNAc-siMCJ after development of NASH prevents its progression. To address the safety of the delivery of siMCJ with GalNAc, we also tested a single dose 3-fold higher than the experimental dose of si393 under physiological conditions for signs of toxicity. No signs of toxicity could be observed 1 week (Supplementary Fig. 17) or 24 h (Supplementary Fig. 18) after administration. Therefore, silencing MCJ in hepatocytes through the delivery of GalNAc-siMCJ shows safety and efficacy for the treatment of steatosis and fibrosis in fatty liver disease.

## Discussion

NAFLD is a complex disease with an alarming increasing incidence around the world. In spite of numerous attempts, the underlying molecular mechanisms responsible for NAFLD remain largely unknown. Numerous pathogenic molecular pathways have been described in the development and progression of NASH. Some of the targets that have been pursued were initially identified as targets for diabetes and other metabolic disorders (e.g., PPAR, FGF-21)^30,31^. De novo lipid synthesis in the liver has been a frequent pathway targeted with the goal of inhibiting and decreasing the production of lipids (e.g., ACC)^30,31^. Blocking the release of lipids from the adipose tissue to prevent their transport to the liver is another approach that has been taken to clinical trials, but this approach can result in weight gain^30,31^. Since fibrosis is the most important predictor of overall and liver-related mortality, different fibrotic targets as well as inflammation (the main trigger of fibrosis) have been extensively explored (e.g., caspases, chemokines, Galactin-3)^30,31^. Interestingly, boosting the catabolism of lipids in the liver mitochondria has received little attention. However, the new thyroid receptor β-agonists, thought to enhance fatty acid β-oxidation in mitochondria, are now in clinical trials as treatment for fatty liver disease^30,31^. Here, we identify a pathway that has not been targeted previously: enhancing mitochondrial ETC activity in hepatocytes.

Two of the main products from fatty acid β-oxidation in the mitochondria are NADH (the substrate reduced to NAD^+^ by Complex I of the ETC) and FADH2 (substrate of Complex II of the ETC) that feed into ETC. However, if the ETC activity is low, the accumulation of NADH and FADH2 within the mitochondria could provide a negative feedback to the β-oxidation process. Thus, increasing Complex I activity and mitochondrial respiration could indirectly enhance the catabolism of fatty acids through fatty acid β-oxidation, but this approach has not been obvious because there are not many known negative regulators of the ETC to be targeted. We have previously identified MCJ as an endogenous negative regulator of Complex I and mitochondrial respiration in multiple cell types including hepatocytes^5–7^. In this study, we demonstrate that the therapeutic inhibition of MCJ expression in vivo enhances fatty acid β-oxidation in the liver in a NASH model. In addition, we also show that the enhanced fatty acid β-oxidation resulting from inhibiting MCJ is due to enhanced Complex I activity. Thus, we identify an alternative pathway to boost β-oxidation and lipid catabolism in the liver by interfering with MCJ expression in hepatocytes.

While increasing fatty acid β-oxidation is a strategy to reduce the accumulation of lipids in the liver and thereby reduce fibrosis, increased β-oxidation is also known to cause increased ROS production in the liver^32–34^ with potential toxic effects. Indeed, treatment with thyroids and thyroid receptor β-agonists can increase ROS levels in the liver, triggering mitophagy as a mechanism to eliminate damaged mitochondria^35^. In contrast, loss of MCJ increases Complex I activity and mitochondrial respiration without increasing mitochondrial ROS production because it also promotes the formation of respiratory complexes (Complex I, III, and IV) in hepatocytes and other cells, and thereby diminishes the risk of electron leak^5–7^. Here we show that in vivo treatment of siMCJ of mice with NASH increases β-oxidation, decreases lipid accumulation in the liver but does not increase ROS production. The JNK pathway has been involved in the initial mechanisms as well as in the progression of NAFLD^36^. Mice lacking JNK1 and JNK2 are largely protected from liver steatosis^37,38^. In addition, mitochondrial ROS are known to activate the JNK pathway, which has been associated with NAFLD^36^, but we have shown that loss of MCJ does not cause activation of JNK in the liver^6^. Thus, unlike thyroid hormone receptor agonists, targeting MCJ in the liver could provide a safer therapeutic option since it prevents the generation of ROS.

Currently, all the therapeutic drugs undergoing clinical trials for NASH are small molecules. Our data show, in contrast, that the use of siRNA to reduce the levels of MCJ in the liver may constitute an alternative therapeutic strategy^16,17^. Following the initial excitement for the use of siRNA for therapeutic purposes, the actual development of successful siRNA therapies has been a long and complicated process. A critical challenge for the efficient development of siRNA-based therapies has been the design of delivery systems that specifically target the tissue of interest. In spite of this, delivery to the liver has produced major success and resulted in some FDA-approved drugs^39^. The most common delivery system for siRNA to the liver has long been LNP and the first LNP-formulated siRNA-based drug (Patisiran) was approved in 2018 for the treatment of hATTR (hereditary transthyretin-mediated) amyloidosis^40^. However, due to potential secondary effects during prolonged treatments, particularly on the immune system, GalNAc was developed as an alternative delivery system for the liver that specifically targets hepatocytes^28,29^. GalNAc-siRNA conjugates are synthesized by incorporating synthetic triantennary GalNAc to a chemically modified siRNA. GalNAc is specifically delivered to hepatocytes due to the presence of the hepatocyte-specific asialoglycoprotein receptor^28,29,41,42^. Unlike LNPs, GalNAc-siRNA conjugates can be delivered by subcutaneous administration (s.c.), which can potentially allow for self-administration. The first GalNAc-conjugated siRNA drug (Givosiran) was FDA approved in 2019 for the treatment of acute hepatic porphyria. There are over 30 clinical trials with different GalNAc-conjugated siRNA to treat multiple clinical indications^43^. Here we show that siMCJ formulated into LNP or GalNAc has efficacy in reducing liver steatosis and fibrosis in multiple NASH mouse models. To date no other siRNA-based drugs have been tested in human clinical trials, therefore GalNAc-siMCJ could be one of the first therapies tested in the future.

Overall, we show that liver-specific delivery systems for MCJ safely reduce steatosis, inflammation, and fibrosis using different mouse models of NAFLD. Thus, treatment with siMCJ could emerge as a potential alternative therapeutic approach for this disease.

## Methods

### Experimental procedures in animals

C57BL/6 J male mice were purchased from Jackson Laboratory (Bar Harbor, ME) or bred at the AAALAC-accredited University of Vermont or CIC bioGUNE from breeding pairs obtained from Jackson Laboratory. MCJ-deficient mice (MCJ KO) were already generated^5^. Leptin receptor mutant (Lepr^db^/J) mice were obtained from Jackson Laboratories. The studies were performed with male mice and experiments were initiated when the mice were between 10 and 15 weeks of age. Animal procedures were approved by the University of Vermont and University of Colorado Animal Care and Use Committee according to the criteria outlined in the Guide for the Care and Use of Laboratory Animals and institutional IACUC review committee. Animal work at CIC bioGUNE was approved by the institutional IACUC and the Competent Authority following Spanish and European regulations.

For fasting studies, all food was removed and tissues were harvested 36 h later.

For studies using the methionine and choline-deficient (MCD) diet model, mice were fed a diet completely deficient in methionine and choline (MCD) (Research Diets A02082002BRi) for either 2 or 3 weeks as indicated.

For studies using the modified methionine-,choline-deficient (mMCD) diet model, mice were fed a choline-deficient diet containing 0.1% methionine with 45 kcal% fat (Research Diets, A06071309i) for 4 or 6 weeks.

For studies using the HF-HFD model, mice were fed a high fat/high fructose diet consisting of a 58% fat diet with sucrose (Research Diets D12331i) in combination with water containing 42 g/L of carbohydrate (55% fructose and 45% sucrose)^23^. After 4 months on the diet, mice were treated weekly with siMCJ formulated with Invivofectamine i.v. or PBS for 5 weeks.

For studies using the CD-HFD model, mice were fed a choline-deficient, high-fat diet (45 kcal% fat, Research Diets, D05010402i) for 4 months prior to the initiation of GalNAc-formulated siMCJ treatment. Mice then received s.c. injections of 10 mg/Kg GalNAc siMCJ381or PBS every 2 weeks for a total of 4 doses.

For the studies using the diet induced obesity (DIO) mouse model and insulin tolerance test (ITT), B6J DIO male mice were purchased from Jackson Laboratory after 13 weeks in the D12492 high fat diet containing 60 kcal% fat. Mice were maintained with the same diet during the treatment. Mice were treated weekly with siMCJ formulated with Invivofectamine i.v. or PBS for only 2 weeks. Insulin tolerance test was performed a week after the last dose of siMCJ. For ITT, blood glucose was determined using a glucometer prior to the injection of insulin (time 0), following by the administration of insulin (0.75 mU/g) and determination of blood glucose after 15, 30, 60, and 90 min using the OneTouch Ultra glucometer and glucose strips.

For the studies using the carbon tetrachloride (CCl_4_) model of liver fibrosis, mice were administered i.p. 0.6 μl/g of CCl_4_ every 3 or 4 days. A group of mice were harvested 2 weeks after the first CCl_4_ dose. After 2 weeks on CCl4, mice were treated with invivofectamine/siMCJ (1.7 mg/Kg) or PBS control group weekly for 3 more weeks. Mice were then euthanized and tissues were harvested.

For treatment with siRNA in vivo, MCJ-specific siRNA (siMCJ) (position 294–312) was designed as previously described^18^. For in vivo delivery using Invivofectamine, siMCJ was complexed with lipid nanoparticle Invivofectamine® 3.0 (ThermoFisher Scientific) according to the manufacturer’s instruction. Mice received weekly i.v. injections of 1.7 mg/Kg of siRNA complexed with invivofectamine. MCJ-specific siRNAs formulated with GalNAc were provided by Alnylam Pharmaceuticals, Inc. GalNAc-siRNAs targeting MCJ were selected using in vitro cell-based activity screens in primary mouse hepatocytes with confirmation of activity in WT mice. The two selected siRNAs (si381 and si393) were conjugated to an *N*-Acetylgalactosamine (GalNAc) for optimized delivery in hepatocytes as previously described^44^. The siRNA duplexes targeting mouse MCJ (Dnajc15) were designed to maximize predicted efficacy and minimize off-target effects using proprietary algorithms (Alnylam). For in vivo screening, mice received a 10 mg/Kg s.c. dose in PBS of different MCJ-specific GalNAc-siRNA selected from the in vitro screening. After 2 weeks, liver expression of DnaJC15 was measured by Taqman RT-PCR using the following assay from ThermoFisher (Mm00481271_m1). GalNAc-si381 or GalNAc-si393 were selected from the in vivo studies and used for treatment of mice on the different diets at 10 mg/Kg in PBS administered s.c. every 2 weeks. Fully chemically-modified GalNAc-siRNA conjugates were employed to confer drug-like properties (Pharmacokinetics, pharmacodynamics, safety). Pattern of 2′-fluoro and 2′-O-methyl modifications were employed to improve in vivo potency^45^. si381 and si393 antisense strands target the following nucleotide sequence of the mouse MCJ transcript (NM_025384.3), respectively (404–424 and 358–378).

### Human samples

Healthy human liver (*n* = 5) samples from Valdecilla Hospital (Santander, Spain) were used as controls. All had histologically healthy liver, BMI <25 Kg/m^2^, normal fasting glucose, cholesterol and TG, normal AST and ALT, and no evidence of viral infections (HBV, HCV, and HIV). Diseased liver samples come from 21 patients with a clinical diagnosis of NAFLD who underwent a liver biopsy with diagnostic purposes in Santa Cristina Hospital (Madrid, Spain). Inclusion criteria for NAFLD patients were based on an alcohol intake lesser than 20 g/day, the presence of biopsy-proven steatosis with/without necroinflammation and/or fibrosis, and no evidence of hepatitis B and/or C virus (HBV and/or HCV, respectively) infection as well as human immunodeficiency virus (HIV) infection. The characteristics of the study groups are described in Table 1. Clinical examination included a detailed interview with special emphasis on both alcohol intake and medications use, history of known diabetes and arterial hypertension, as well as measurements of weight, height, blood pressure, and waist and hip perimeters. Body mass index (BMI) was calculated as weight (Kg) divided by height (m) squared. Fasting blood samples were obtained and used to measure alanine and aspartate transaminases (ALT and AST, respectively), GGT, total cholesterol, HDL-cholesterol, triglyceride, glucose, HbA1c, and insulin. Insulin resistance was calculated using the homeostatic model assessment (HOMA-IR) index^1^. Hepatic histopathological analysis was performed according to the scoring system of Kleiner et al.^2^. Four histopathological features were semi-quantitatively evaluated: grade of steatosis (0, <5%; 1, 5%–30%; 2, >30–60%; 3, >60%), lobular inflammation (0, no inflammatory foci; 1, <2 inflammatory foci per 200x field; 2, 2–4 inflammatory foci per 200x field; 3, >4 inflammatory foci per 200x field), hepatocellular ballooning (0, none; 1, few balloon cells; 2, many cells/prominent ballooning), and stage of fibrosis (from 0, none to 4, cirrhosis). Simple steatosis was defined as the presence of at least 5% of steatotic hepatocytes with or without mild lobular or portal inflammation but in the absence of features of hepatocellular injury (ballooning, apoptosis, or necrosis) and fibrosis. On the other hand, minimal criteria for the histological diagnosis of definite NASH included the combined presence of grade 1 steatosis, hepatocellular injury, and lobular inflammation with or without fibrosis. The study was performed in agreement with the Declaration of Helsinki, and with local and national laws. The Human Ethics Committee of Santa Cristina Hospital, the Comité Consultatif de Protection des Personnes dans la Recherche Biomédicale de Nice or the Human Ethics Committee of Valdecilla Hospital approved the study procedures, and written informed consent was obtained from all patients before inclusion in the study.

**Table 1:** Characterization of NAFLD patients.

	NAFLD patients
Number of patients	21
Age (years)	50.2 ± 15.2
BMI (kg/m²)	29.6 ± 3.9
Obesity, *n* (%)	7 [33.3%]
Gender (Female/Male)	17/4
ALT (IU/L)	24.2 ± 16.8
AST (IU/L)	20.4 ± 7.6
GGT (IU/L)	49.9 ± 50.2
HOMA-IR	2.7 ± 1.9
Insulin resistance, *n* (%)	11 [52.4%]
Total cholesterol (mg/dl)	212 ± 46.1
HDL-cholesterol (mg/dl)	48.4 ± 16.2
Triglyceride (mg/dl)	159.5 ± 72.3
Liver steatosis grade: 0/1/2/3 (n)	0/15/5/1
NASH, *n* (%)	3 [14.3%]
Grade of liver fibrosis: 0/1/2/3/4 (n)	19/2/0/0/0

*BMI* body mass index, *ALT* alanine aminotransferase, *AST* aspartate amino transferase, *GGT* gamma glutamyl transferase, *HOMA-IR* homeostatic model assessment of Insulin resistance, *HDL* high density lipoprotein, *NASH* non-alcoholic steatohepatitis.

### Histology

To perform H&E and Picro Sirius Red staining, paraffin-embedded liver samples were sectioned, dewaxed with a xylene substitute (HS-202, Histoclear; National Diagnostics, Atlanta, GA), and hydrated. Sections were stained for 5 min with Harry’s hematoxylin (HHS128-4L; Sigma) and for 15 min with aqueous eosin (HT110232-1L; Sigma) for H&E staining or with 0.01% Fast green FCF in saturated picric acid for 15 min and 0.04% Fast green For Coloring Food/0.1% Sirius red in saturated picric acid for 15 min for Sirius red staining. Samples were dehydrated and cleared with Histoclear. Finally, sections were mounted in DPX mounting media (06522, 500 mL; Sigma).

To perform Sudan III staining ornithine carbamyl transferase–embedded frozen samples were sectioned, cleared with 60% isopropanol, and stained with Sudan III solution (0.5% in isopropanol Sudan III Panreac) for 1 h and finally cleared with 60% isopropanol. Sections were counterstained with Mayer hematoxylin (MHS32-1L; Sigma) and mounted in aqueous mounting medium for lipid quantification.

To perform Oil Red O staining tissue sections from frozen tissue were stained with 0.2% Oil Red O and counterstained with hematoxylin.

To perform immunostaining in liver section, F4/80 samples were unmasked with proteinase K during 15 min at room temperature and MCJ samples with citrate buffer pH 6.0 during 20 min at 97 °C. Endogenous peroxidase activity was blocked for 10 min with 3% hydrogen peroxide, then sections were blocked with 5% normal goat serum for 30 min and incubated with F4/80 (1:50, 1 h at 37 °C, MCA497BB; Bio-Rad, Hercules, CA) or MCJ (B0027R BioMosaics) (1.100, overnight at 4 °C) followed by 30 min with anti-rat or anti-mouse reagents. Colorimetric detection was completed with Vector Vip purple substrate (sk-4600; Vector). Slides were counterstained with Mayer Hematoxylin (MHS32-1L; Sigma), and finally samples were dehydrated, cleared, and mounted in DPX mounting media (06522-500 mL; Sigma).

For the analysis, images from liver sections stained H&E, Picro Sirius Red, Sudam III, or Oil Red Oil were taken with an upright light microscope (Zeiss, Germany), or slides were scanned using the Leica-Aperio Versa (University of Vermont Imaging Facility). The average sum of intensities and stained area percentage of each sample was calculated using FRIDA software (http://bui3.win.ad.jhu.edu/frida/, John Hopkins University). No manipulation of the histological microscopy images was performed.

### De novo lipogenesis analysis in liver

De novo lipogenesis was performed as previously described^3^ with slight modifications. In brief, freshly isolated tissue slices (40 mg) were incubated in high glucose DMEM with insulin (150 nM) and [H^3^] Acetic acid 20 µCi/ml for 4 h. Tissue slices were washed five times in cold PBS, homogenized in PBS and lipids were extracted and separated by TLC^4,5^, each lipid was scraped and the radioactivity was measured in a scintillation counter.

### β-oxidation analysis

Beta oxidation was assessed as follows^6,7^. Fresh liver pieces were homogenized using a Potter homogenizer (5 strokes) in cold buffer (25 mM Tris-HCl, 500 nM sucrose, 1 mM EDTA-Na2 pH 7,4) and sonicated for 10 s. Then, the homogenates were centrifuged at 500 × *g* for 10 min at 4 °C. Approximately 500 µg of protein from the homogenate supernatants was used for the assay in a volume of 200 µl. The reaction started by adding 400 µl of assay mixture containing 0.5 µCi/ml [1-^14^C] palmitic acid to the samples and was incubated for 1 h at 37 °C in microfuge tubes with a Whatman paper circle in the cap. The reaction was stopped by adding 300 µl of 3 M perchloric acid and 1 M NaOH was added to impregnate the Whatman cap. After 2 hours the Whatman caps were retired and the radioactivity associated was measured in a scintillation counter. The microfuge tubes were centrifugated at 21,000 × *g* for 10 min at 4 °C. 400 µl of the supernatants were collected and the radioactivity was counted in a scintillation counter. The supernatant contained the ASM and the Whatman caps captured the released CO_2_.

### Serum analysis

Serum TGA were measured using a commercially available kit Triglycerides Liquid Mono (Krotest Laboratorios). Serum ketone bodies were measured using a commercially available kit from Wako Chemicals (Richmond, VA). Serum albumin and urea were measured using BioVision’s Albumin Assay Kit and Urea Assay Kit II, respectively (Milpitas, CA). Aspartate aminotransferase (AST) and alanine aminotransferase (ALT) were measured by multiple-point rate reflectance spectrophotometry on a Orhto Vitros 5600 analyzer. Serum insulin levels were determined using the Insulin (Mouse) ELISA kit (BioVision).

### Isolation and culture of primary hepatocytes

Primary hepatocytes were isolated from male C57BL/6 WT mice via collagenase perfusion^8^. For in vitro silencing, WT primary hepatocytes were transfected with 100 nM MCJ siRNA using Jetprime reagent (Polyplus). Controls were transfected with an unrelated siRNA (Qiagen). For BODIPY staining, primary hepatocytes were incubated with 400 μM oleic acid and the complex I inhibitor Rotenone (Sigma-Aldrich) (1 μM) for the indicated times. Hepatocytes in culture were incubated with BODIPY 493/503 (Molecular Probes) at 1 mg/ml during 30 min prior to fixation (4% paraformaldehyde). Imaging was performed with an Axioimager D1 microscope and quantification of lipid bodies was performed using Frida Software.

### Western blot analysis

Total protein and mitochondrial fraction protein from primary hepatocytes and liver tissue were resolved on sodium dodecyl sulfate-polyacrylamide gels and transferred to nitrocellulose or polyvinylidene difluoride (PVDF) membranes. Membranes were incubated with anti-mouse MCJ^9^ or antimouse GAPDH (Santa Cruz, Abcam) overnight. Primary antibodies were detected with anti-rabbit-IgG-HRP (Cell Signaling, Jackson Laboratory) and anti-mouse IgG-HRP (Santa Cruz Biotechnology) and signal developed with chemiluminescence (KPL) on an Amersham Imager 600.

### RNA isolation and quantitative real-time polymerase chain reaction (RT-PCR)

Total RNA was isolated with Trizol (Invitrogen). Total RNA (1–2 μg) was treated with DNase (Invitrogen) and reverse transcribed into cDNA using M-MLV Reverse Transcriptase (Invitrogen). Quantitative real -time PCR (RT-PCR) was performed using SYBR^®^ Select Master Mix (Applied Biosystems) and the Viia 7 Real-Time RT-PCR System (Applied Biosystems). The Ct values were extrapolated to a standard curve, and data was then normalized to the house-keeping expression (GAPDH). For real time RT-PCR of Acta2, we used the assay on demand primers and probe #Mm00725412 from ThermoFisher. Primers used for the rest of the other analyses are shown in Supplementary Table 1.

### Mitochondrial respiration analysis

Liver mitochondrial respiration was measured at 37 °C by high-resolution respirometry using the Seahorse Bioscience XF24-3 Extracellular Flux Analyzer. For the measurement of the OCR, as the rate change of dissolved O_2_, liver mitochondria were isolated and plated in a XF24 cell culture microplate (Seahorse Bioscience), 5 μg per well as indicated^10^. Mitochondria were incubated in a media containing substrates for both complexes I and II: Glutamate (10 mM), Malate (2 mM), and Succinate (10 mM). After an OCR baseline measurement, sequential injections through ports in the XF Assay cartridges of pharmacologic inhibitors: ADP (4 mM) (state 3 respiration), Oligomycin (3 μM), an inhibitor of ATP synthase, which allows a measurement of ATP-coupled oxygen consumption through oxidative phosphorylation (OXPHOS); carbonyl cyanide 4-trifluoromethoxy-phenylhydrazone (FCCP) (4 μM), an uncoupling agent that allows maximum electron transport, and therefore a measurement of maximum OXPHOS respiration capacity; and finally Antimycin A (4 μM) + Rotenone (2 μM), mitochondrial complex I and III inhibitors respectively, were performed and changes in OCR were analyzed. The normalized data were expressed as pmol of O_2_ per minute or milli-pH units (mpH) per minute, per viability measured by MTT assay.

### MDH2 activity

MDH2 activity was measured in liver extracts using the recommendation from the kit from Abcam ab119693.

### ATP level determination

The levels of ATP in liver mitochondria were determined using the ATPlite luminescence ATP detection assay system (PerkinElmer) by following the recommendations from the manufacturer. Mitochondrial fractions were obtained using the Mitochondrial Fractionation Kit (ActiveMotif) for hepatic tissue.

### ROS production analysis

For GSH quantification, liver extracts were analyzed with a UPLC system (Acquity, Waters, Manchester) coupled to a Time of Flight mass spectrometer (ToF MS, SYNAPT G2, Waters). A 2.1 × 100 mm, 1.7 μm BEH amide column (Waters), thermostated at 40 °C, was used to separate the analytes before entering the MS. Solvent A (aqueous phase) consisted of 99.5% water, 0.5% formic acid, and 20 mM ammonium formate while solvent B (organic phase) consisted of 29.5% water, 70% MeCN, 0.5% formic acid, and 1 mM ammonium formate. The extracted ion trace was obtained for GSH (m/z = 308.0916) in a 20 mDa window and subsequently smoothed (2 points, 2 iterations) and integrated with QuanLynx software (Waters, Manchester). Concentrations were converted into amount of analyte per mg liver tissue.

To evaluate lipid peroxidation in liver as marker of oxidative stress^21,22^, liver sections were immunostained for 4-HNE (Abcam, UK) as recommended by the manufacturer (Abcam). Immunostained images were visualized on an Axioimager D1 microscope, and the results were analyzed using the Frida software.

### Statistical analysis

Statistical significance was determined by one-way analysis of variance or Student’s *t* test analysis, otherwise specified. All error bars represent standard deviation (SD), except when specified.

Comparison of DNAJC15 promoter CpG methylation with gene expression. DNA methylation IDAT files for 50 TCGA healthy liver samples^46^ were downloaded from the Genomic Data Commons (gdc.cancer.gov) and pre-processed using in-house pipelines. The pre-processing/filtering procedure left 451,561 CpGs in 47 noncancer liver tissues. The data set was further restricted to 40 subjects with matched RNA-seq data. The relation of the 3 *DNAJC15* CpGs (cg14729962, cg09677945, cg15988970) and gene expression was determined by linear regression implemented in R version 3.5.1.

Comparison of DNAJC15 methylation in NAFLD to normal human liver. MCJ/DnaJC15 DNA methylation profiles from NAFLD patients and healthy human liver were publicly available in six GEO, (www.ncbi.nlm.nih.gov/geo) data sets listed in Table 2.

**Table 2:** Gene expression omnibus data sets.

Data set [references]	NAFLD number^a^	Healthy number
GSE48325^47,48^^,b^	14	18
GSE49542^49^	59	0
GSE61258^48^	14	26
GSE61278^50^	0	52
GSE65057^51^	0	7
GSE69852^52^	0	3
Total	87	106

^a^Number of subjects in each set.

^b^Reference number.

Two-tailed Wilcoxon’s rank sum tests were used to compare methylation of the three CpGs of interest (cg14729962, cg09677945, cg15988970)^15^ between liver tissue of NAFLD and healthy subjects. The total sample sizes for statistical comparisons were: 193, 190, and 141 for each of the above CpGs listed; cg09677945 was missing in GSE69852 (*n* = 3 healthy), and cg15988970 was missing in GSE61278 (*n* = 52 healthy). All statistical analysis and data visualization were performed in R version 3.5.1.

### Reporting summary

Further information on research design is available in the Nature Research Reporting Summary linked to this article.

## Supplementary information

Supplementary Information

Reporting Summary

## Data Availability

The authors declare that all the data supporting the findings of this study are available within the paper and its supplementary information files. The publicly available datasets analyzed to assess *DNAJC15* methylation profiles during the current study are available in the Gene Expression Omnibus repository with the accession codes GSE48325, GSE49542, GSE61258, GSE61278, GSE65057, and GSE69852. Source data are provided with this paper.

## Source data

Source Data File
