# Supplementary material for: Silencing hepatic MCJ attenuates non-alcoholic fatty liver disease (NAFLD) by increasing mitochondrial fatty acid oxidation
Source: Nat Commun. 2020 Jul 3;11:3360. doi: 10.1038/s41467-020-16991-2 (PMC7334216; doi:10.1038/s41467-020-16991-2)
Supplement: Supplementary file 1 — Supplementary Information [file 41467_2020_16991_MOESM1_ESM.pdf]

Silencing hepatic MCJ attenuates non-alcoholic fatty liver disease (NAFLD) by increasing mitochondrial fatty acid oxidation

Barbier-Torres et al

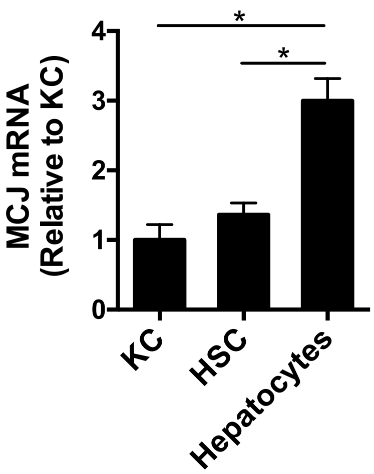

Supplementary Fig. 1

Supplementary Figure 1. MCJ expression in isolated Kupffer cells (KC), hepatic stellate cells (HSC) and hepatocytes from liver of wildtype mice, as determined by real time RT-PCR and presented as fold-induction relative to Kupffer cells. *denotes $p<0.05$, as determined by one-way ANOVA. Error bars show standard error (SE).

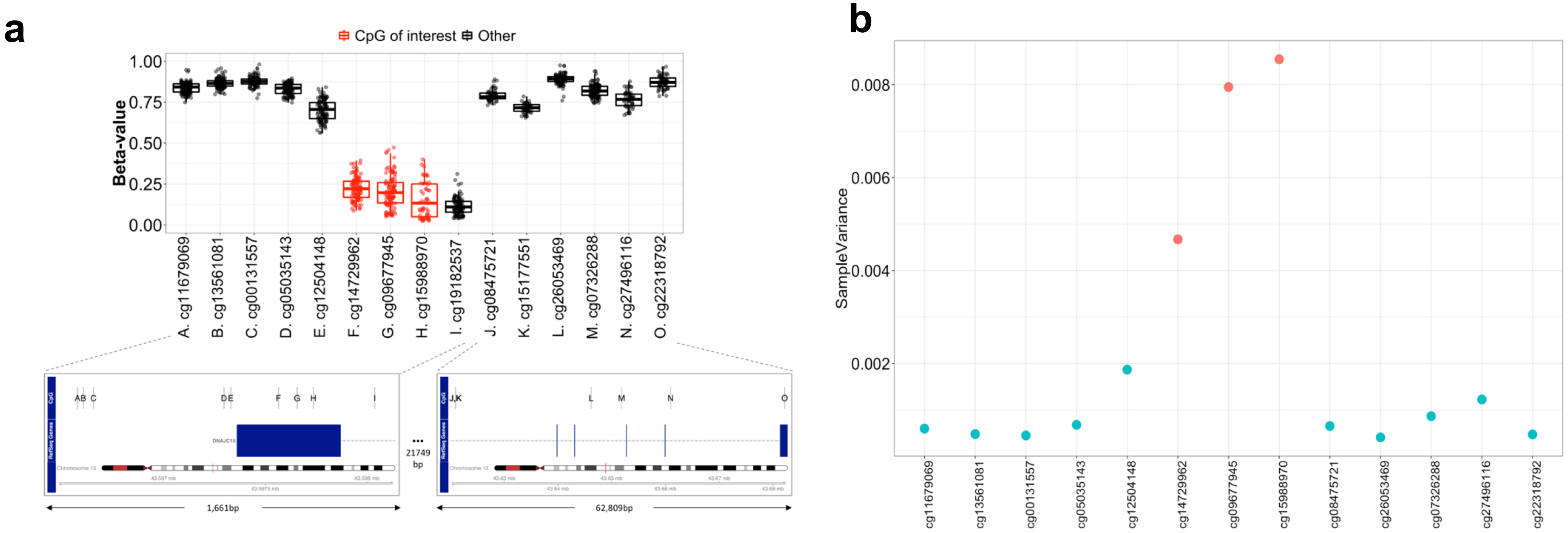

Supplementary Figure 2. Demethylation of 3 specific CpG correlated with increased *DNAJC15* expression. (**a**) Distribution of methylation beta-values of all *DNAJC15* CpGs in healthy human liver. Insets show the genomic position of each CpG along the *DNAJC15* locus in the human hg19 genome assembly. (**b**) Levels of inter-sample variance of all *DNAJC15* CpGs sorted by genomic position in human healthy liver. Results show that the 3 sites have the highest variability relative to other *DNAJC15* methylation loci.

Supplementary Fig. 2

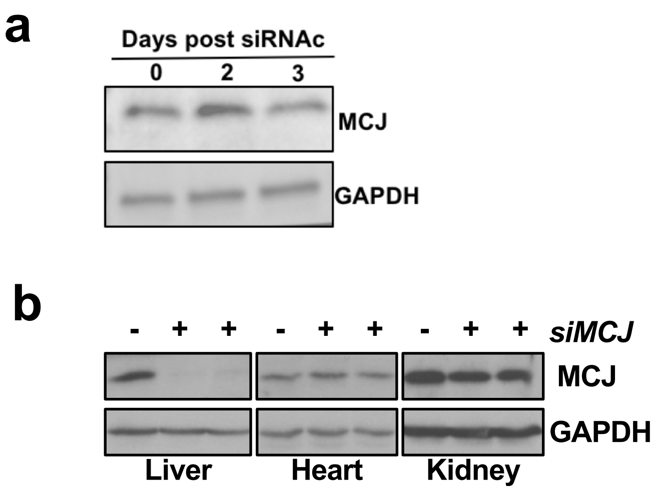

Supplementary Figure 3. **Silencing of MCJ expression selectively in the liver by administration of Invivofectamine-formulated siMCJ**. (**a**) WT mice received a dose of a control siRNA (siRNAc) with Invivofectamine and livers were harvested 2 or 3 days post-injection or prior to administration (time 0). MCJ levels in the liver were determined by Western blot analysis. (**b**) WT mice received a single i.v. dose of Invivofectamine-siMCJ or PBS. Livers, hearts, and kidneys were harvested 2 days after injection and MCJ levels were examined by Western blot analysis. GAPDH was used as a loading control. Each lane represents one mouse.

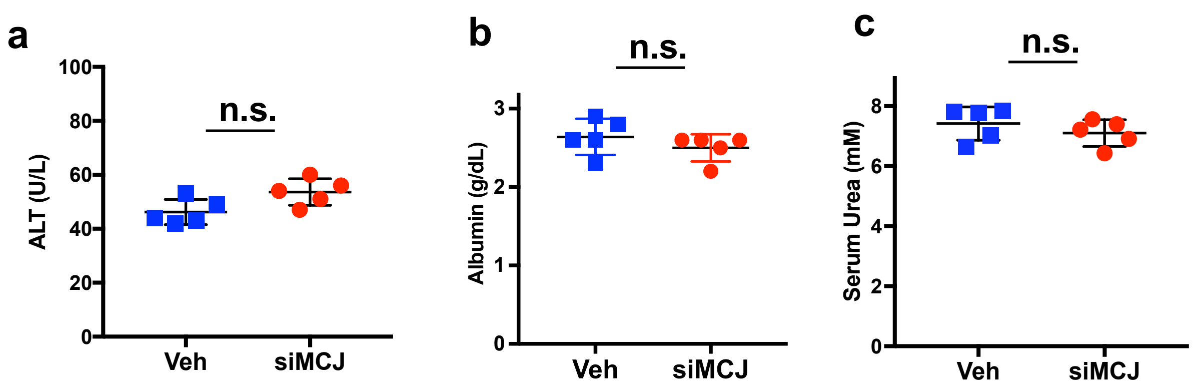

Supplementary Figure 4. Toxicity studies for Invivofectamine-formulated siMCJ. Mice received a single i.v. dose (5-fold higher than the experimental dose, 9 mg/Kg) of Invivofectamine-formulated siMCJ (n=5) or PBS (n=5). After one week, mice were harvested for collection of serum. Serum levels of (**a**) alanine aminotransferase (ALT), (**b**) albumin, and (**c**) urea are shown. n.s. denotes not-statistically significant ($p>0.05$) as determined by t-test analysis. Error bars show standard deviation (SD) in all panels

Supplementary of Fig. 4

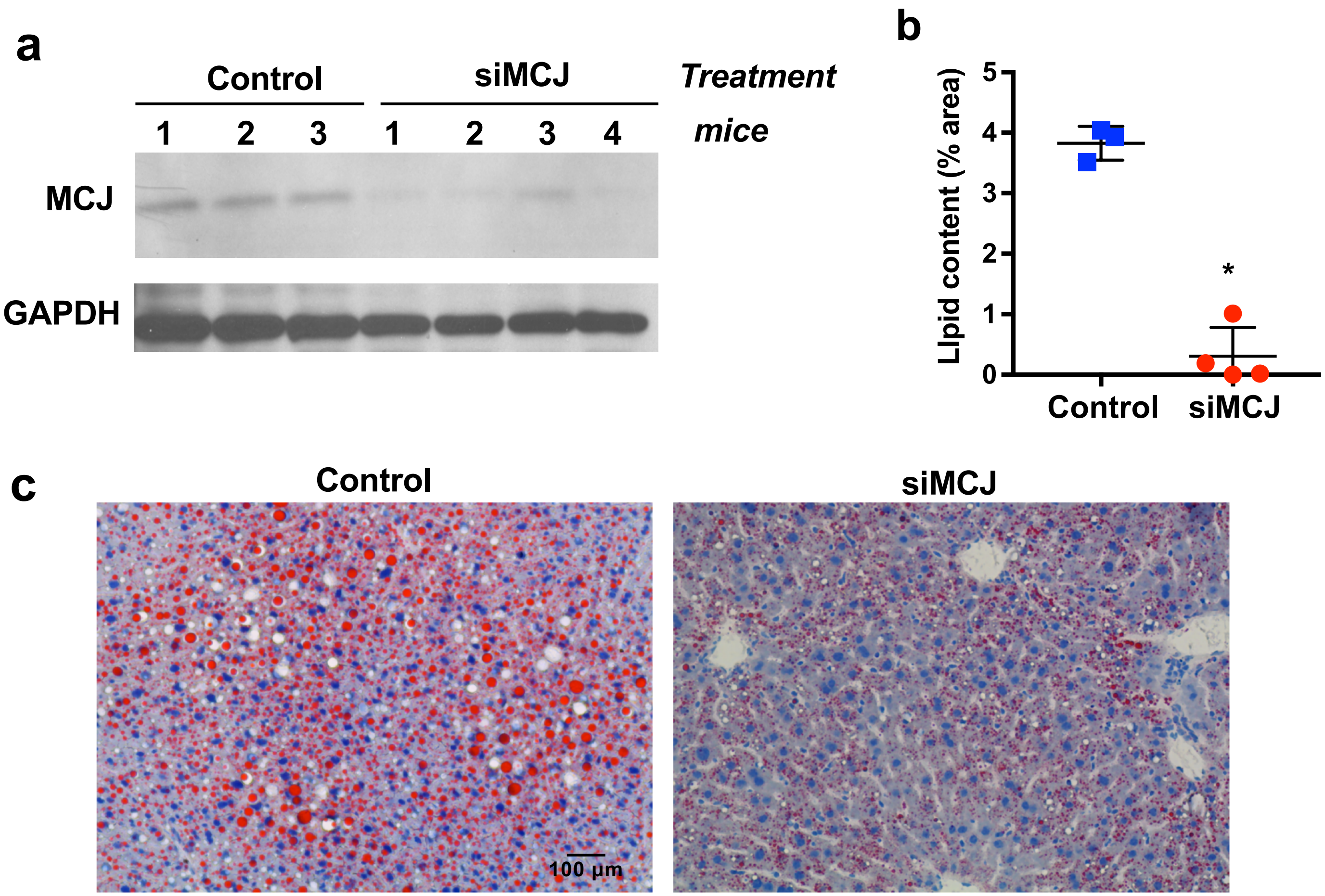

Supplementary Figure 5. Invivofectamine-siMCJ administration largely prevents lipid accumulation in the liver following fasting. Mice received a single i.v. dose of Invivofectamine-formulated siMCJ (n=4) or PBS (Control) (n=3). 18 h after injection, mice were deprived of food and tissues were harvested following 36 h of fasting. (**a**) Expression of MCJ in the livers by Western blot analysis. (**b**) Quantitation of liver lipids by Oil Red O staining of liver sections. (**c**) Representative images of liver sections stained with Oil Red O. *denotes $p<0.05$, as determined by t-test. Error bars show standard deviation (SD).

Supplementary Fig. 5.

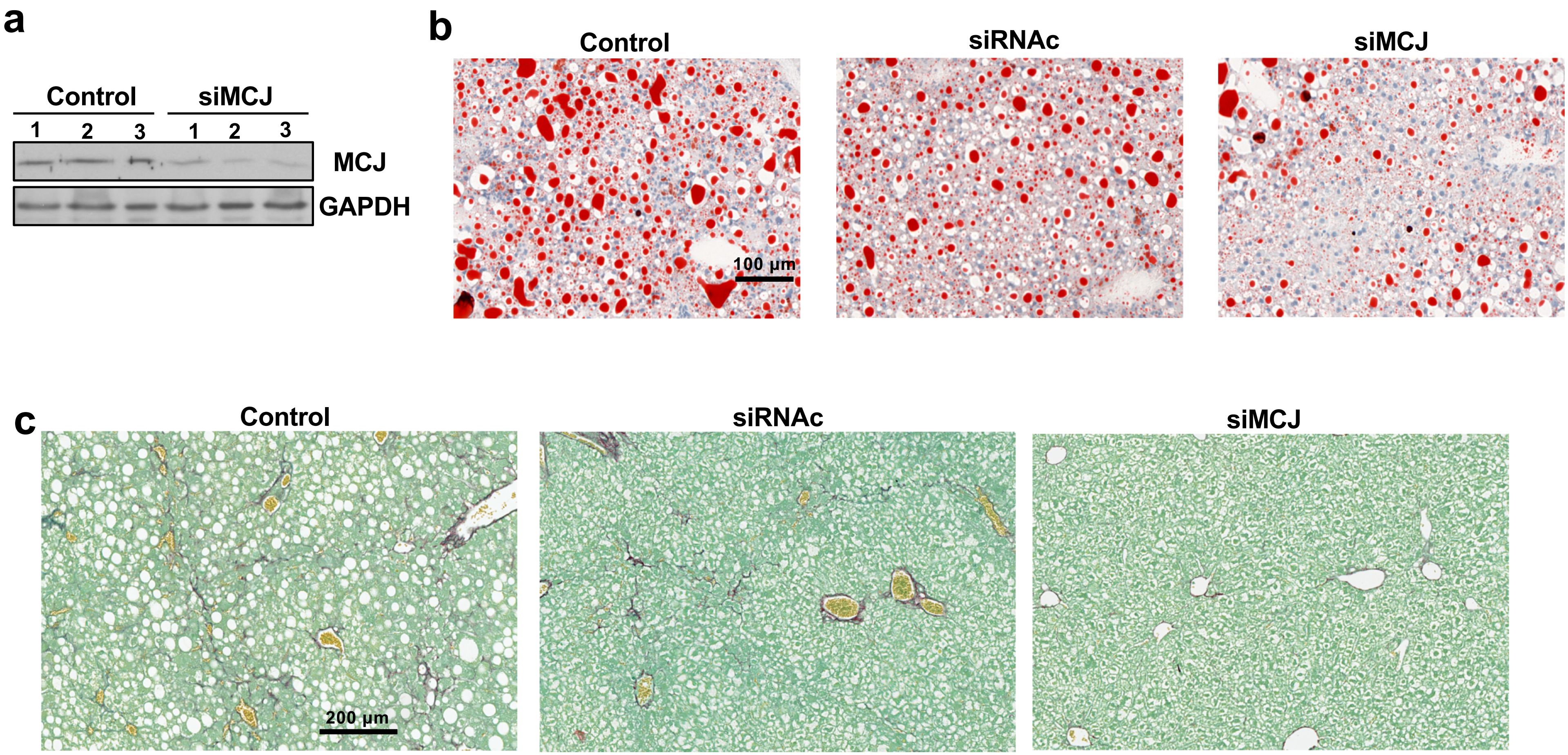

Supplementary Figure 6. Invivofectamine-siMCJ administration markedly reduces lipid accumulation and fibrosis in the liver in the MCD diet model of fatty liver disease. Mice received one i.v. dose of Invivofectamine-formulated siMCJ, Invivofectamine-formulated control siRNA (siRNAc), or PBS (Control) at the time they were placed on MCD diet and a second dose after one week on the diet. Tissues were harvested one week later. (**a**) Expression of MCJ in the livers by Western blot analysis (each lane represents a mouse). (**b**) Representative images of Oil Red O staining of liver sections. (**c**) Representative images of Picro Sirius Red staining of liver sections.

Supplementary Fig. 6

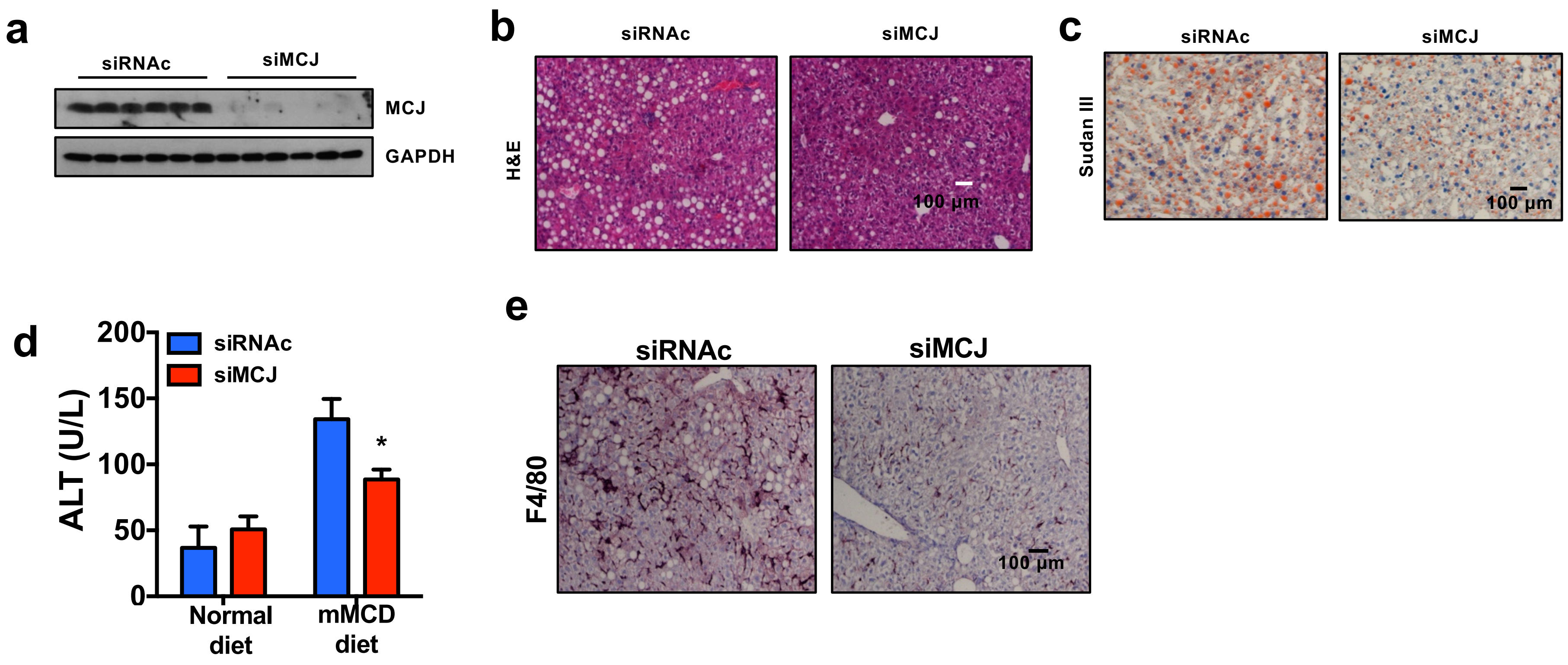

Supplementary Figure 7. Invivofectamine-siMCJ treatment reduces liver lipid accumulation and fibrosis in the mMCD diet model of fatty liver disease. Mice were placed on the modified MCD (mMCD) diet and after one week on the diet they were treated weekly with Invivofectamine-siMCJ (n=6) or siRNAc (n=6) for 3 more weeks. Tissues were harvested one week after the last dose. (**a**) MCJ expression by Western blot analysis. Each lane represents one mouse. (**b**) Representative images of H&E staining of liver sections. (**c**) Representative images of liver lipids by Sudan III staining. (**d**) Serum ALT levels. (**e**) F4/80 immunostaining of liver sections. *denotes $p < 0.05$ siMCJ *vs* siRNAc as determined by t-test analysis. Error bars show standard error (SE).

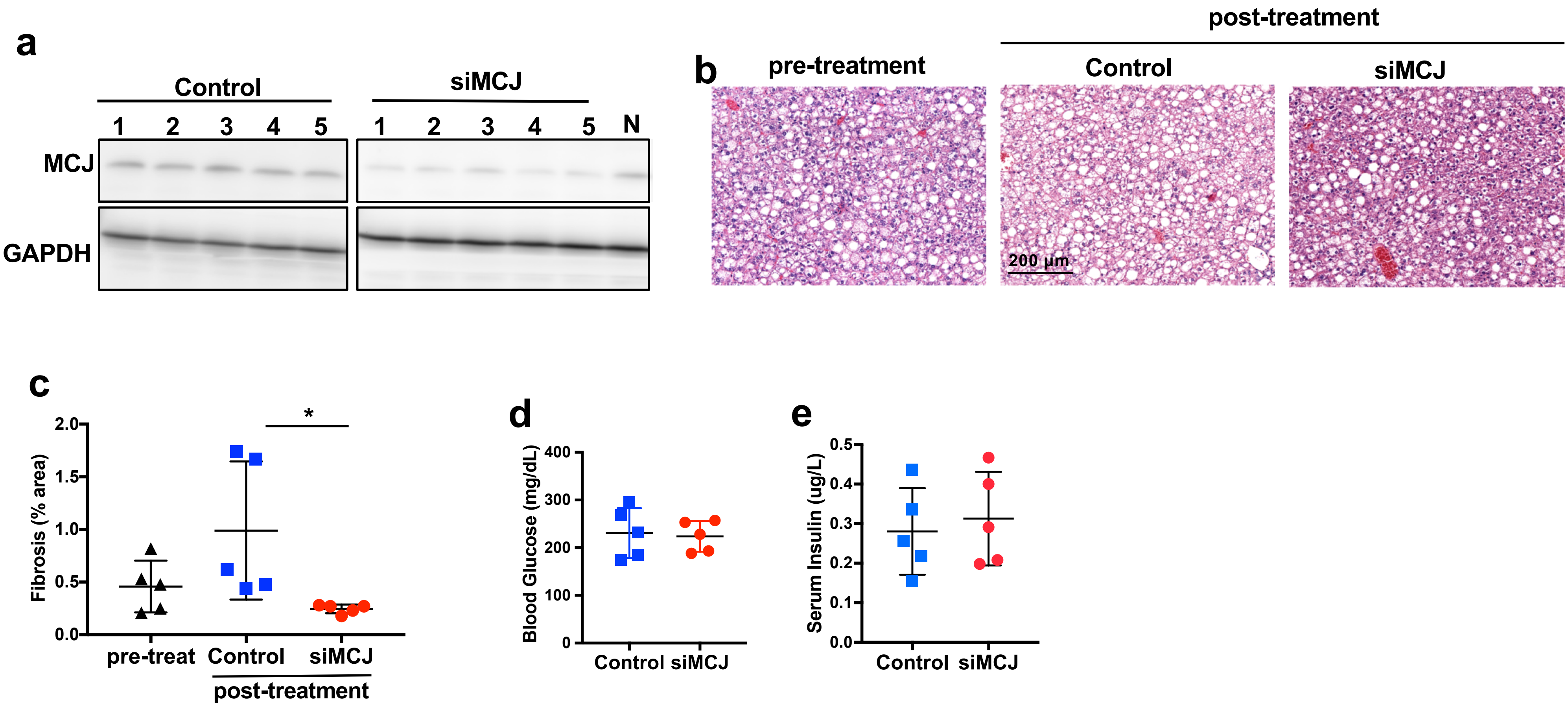

Supplementary Figure 8. Invivofectamine-siMCJ treatment reduces liver lipid accumulation and fibrosis in the HF-HFD model of fatty liver disease. Mice were placed on the high-fat high-fructose diet (HF-HFD) for 4 months prior to the initiation of the treatment (pre-treatment) with Invivofectamine-siMCJ (n=5) or PBS (Control) (n=5) weekly for 5 weeks. Tissues were harvested one week after the last dose. (**a**) Expression of MCJ in the livers by Western blot analysis. Each lane represents one mouse. (**b**) Representative images of H&E staining of liver sections of mice prior to the initiation of the treatment (pre-treatment), and PBS-treated mice or siMCJ-treated mice after 6 weeks. (**c**) Quantification of liver fibrosis on Picro Sirius Red stained-liver sections. (**d**) Blood glucose levels in fed-stage as determined by glucometer. (**e**) Serum insulin levels in fed-stage as determined by ELISA. *denotes $p<0.05$, as determined by one-way ANOVA. Error bars show standard deviation (SD) in all panels.

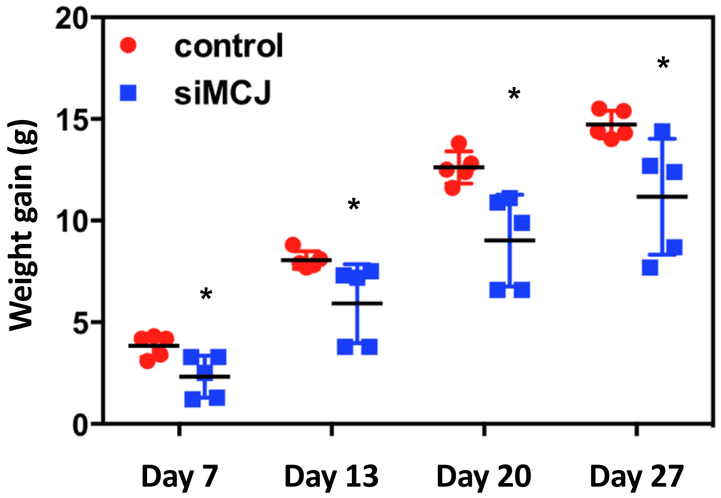

Supplementary Fig. 9. LepR^{db} mice received weekly administrations of invivofectamine-formulated siMCJ or vehicle starting a 5 weeks of age. Shown is the weight gain at the day indicated compared with the starting weight (day 0) for individual mice. * denotes p<0.5, as determined by unpaired t-test). Error bars show standard deviation (SD). .

Supplementary Fig. S9

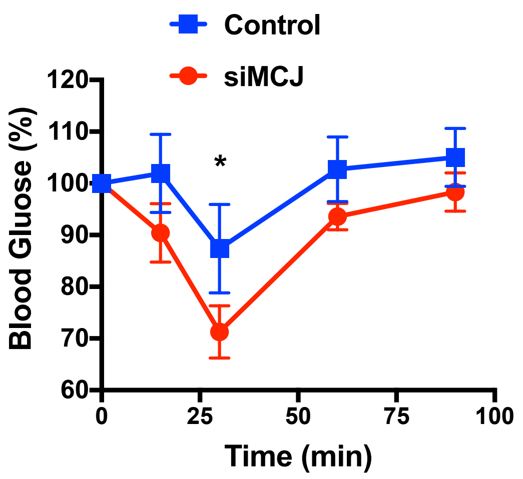

Supplementary Fig. 10. 15 weeks DIO mice received two weekly administrations of invivofectamine-formulated siMCJ (n=8) or vehicle (n=7) starting a 5 weeks of age. A week after the second dose, food was removed 2 hours prior to administration of insulin (0.75 mU/g) and glucose levels in blood were tested using a glucometer at 0 (prior to insulin administration), 15, 30, 60 and 90 min after insulin administration. * denotes $p < 0.05$, at 30 min as determined by two-way ANOVA with Fisher's post hoc comparison. Error bars show standard error (SE).

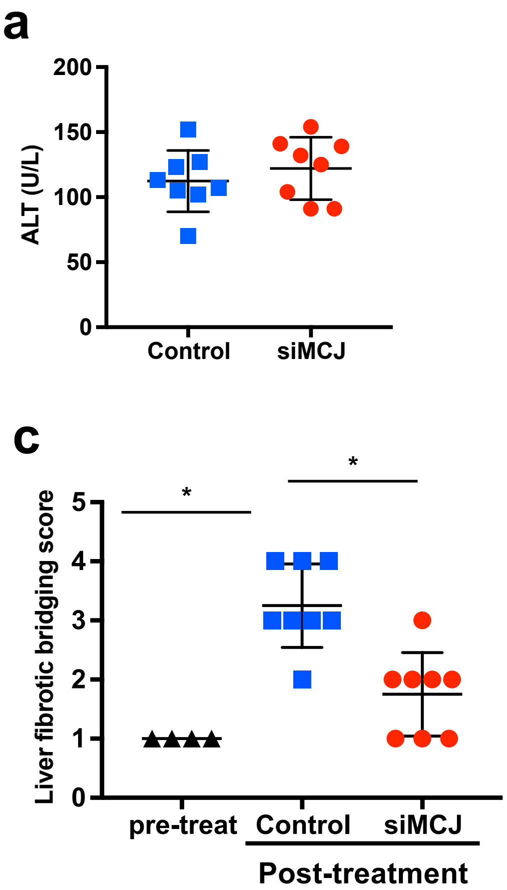

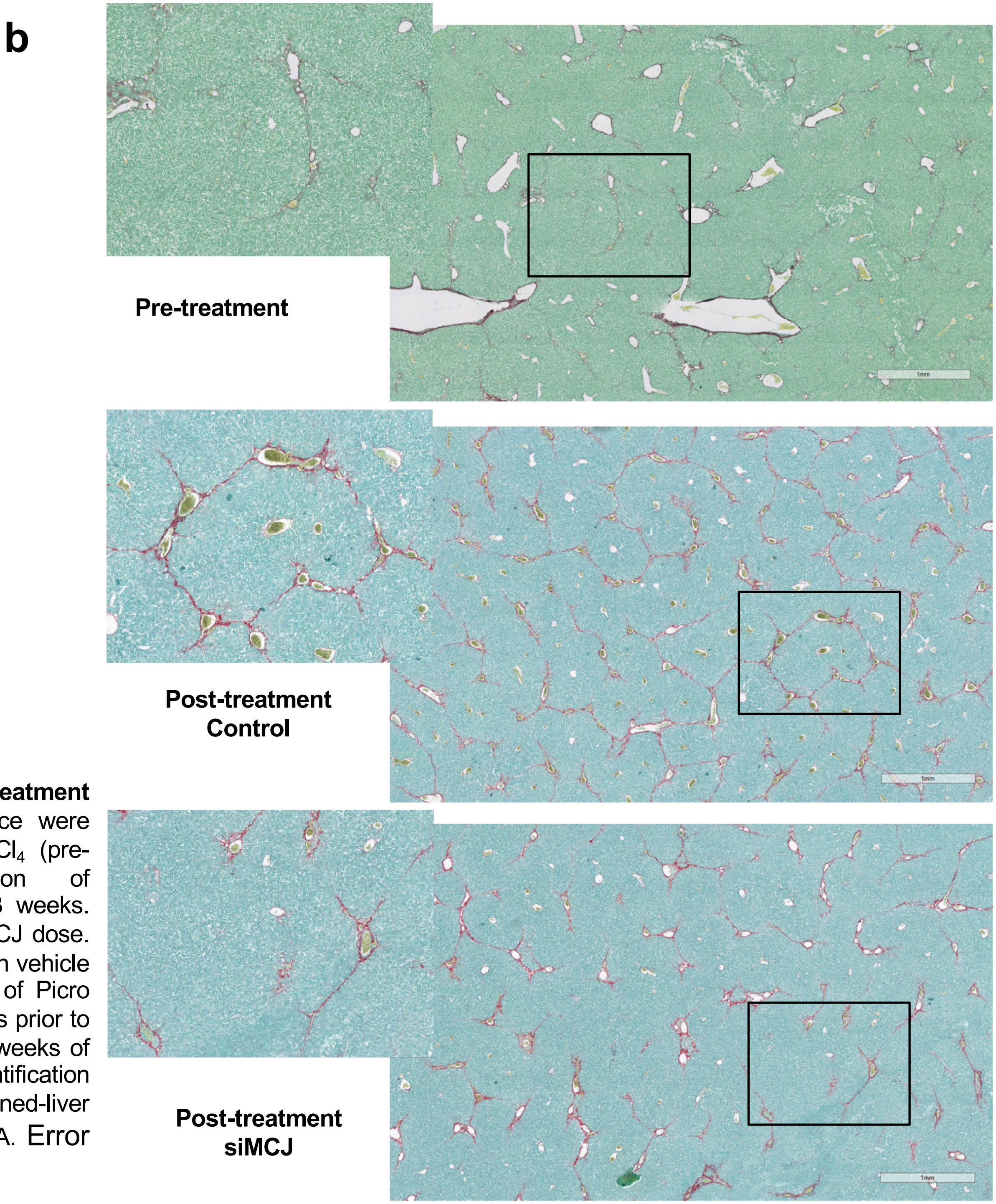

Supplementary Figure 11. Invivofectamine-siMCJ treatment reduces liver fibrosis in the CCl_4 model of NASH. Mice were administered CCl_4 3 times per week. After 2 weeks on CCl_4 (pre-treatment) they received a weekly administration of Invivofectamine/siMCJ (n=8) or vehicle (Control) (n=8) for 3 weeks. Tissues and sera were harvested one week after the last siMCJ dose. (**a**) ALT levels in serum in mice on CCl_4 for 5 weeks treated with vehicle (Control) or siMCJ for 3 weeks. (**b**) Representative images of Picro Sirius Red staining of liver sections of mice on CCl_4 for 2 weeks prior to the initiation of the treatment (pre-treatment), or mice after 3 weeks of treatment (post-treatment) on CCl_4 for 5 weeks total. (**c**) Quantification of fibrotic bridging in the liver based on Picro Sirius Red stained-liver sections. *denotes $p<0.05$, as determined by one-way ANOVA. Error bars show standard deviation (SD) in all panels.

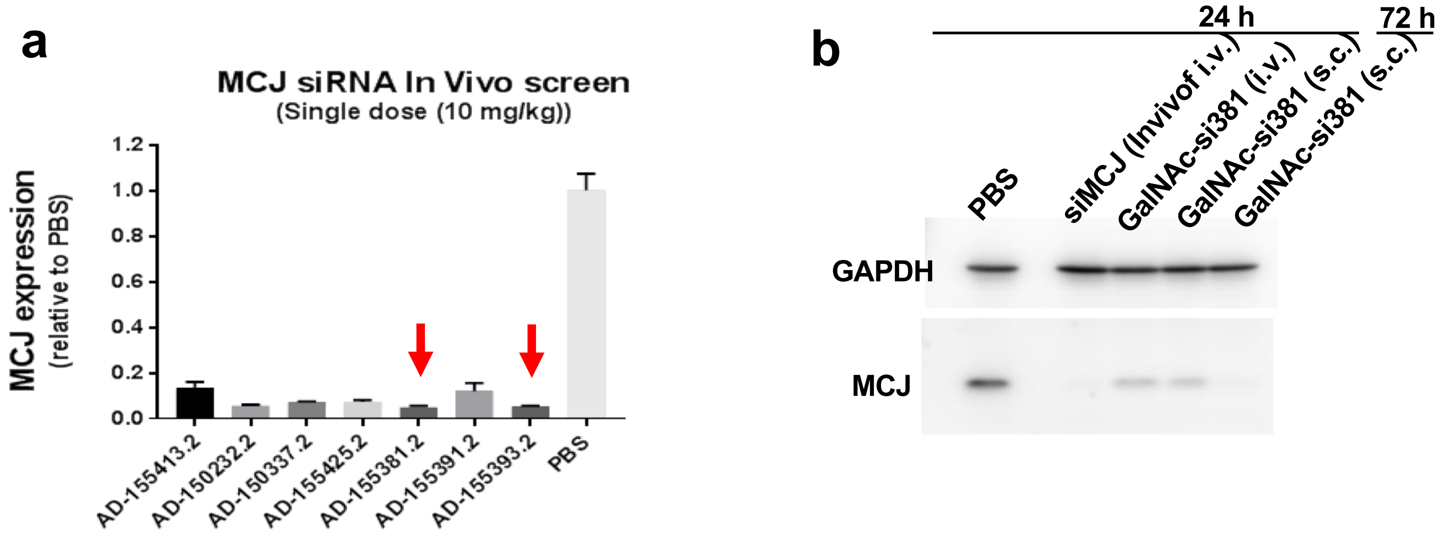

Supplementary Figure 12. GalNAc-formulated MCJ siRNA administration efficiently reduces MCJ expression *in vivo*. (**a**) Mice received a single s.c. dose (10 mg/Kg) of GalNAc-formulated MCJ-specific siRNAs or PBS. Relative *Dnajc15* expression in the liver was analyzed 14 days post-administration by real time RT-PCR, using the levels in PBS-treated mice as reference. Two MCJ-specific siRNAs (si381, si393, arrows) were chosen for further study. (**b**) Mice received an i.v. dose of Invivofectamine-formulated siMCJ (1.7 mg/Kg), an i.v. dose of GalNAc-si381 (10 mg/Kg), a s.c. dose of GalNAc-si381 (10 mg/Kg) or PBS and livers were harvested 24 or 72 h later. MCJ expression in the liver was examined by Western blot analysis. GAPDH was used as a loading control.

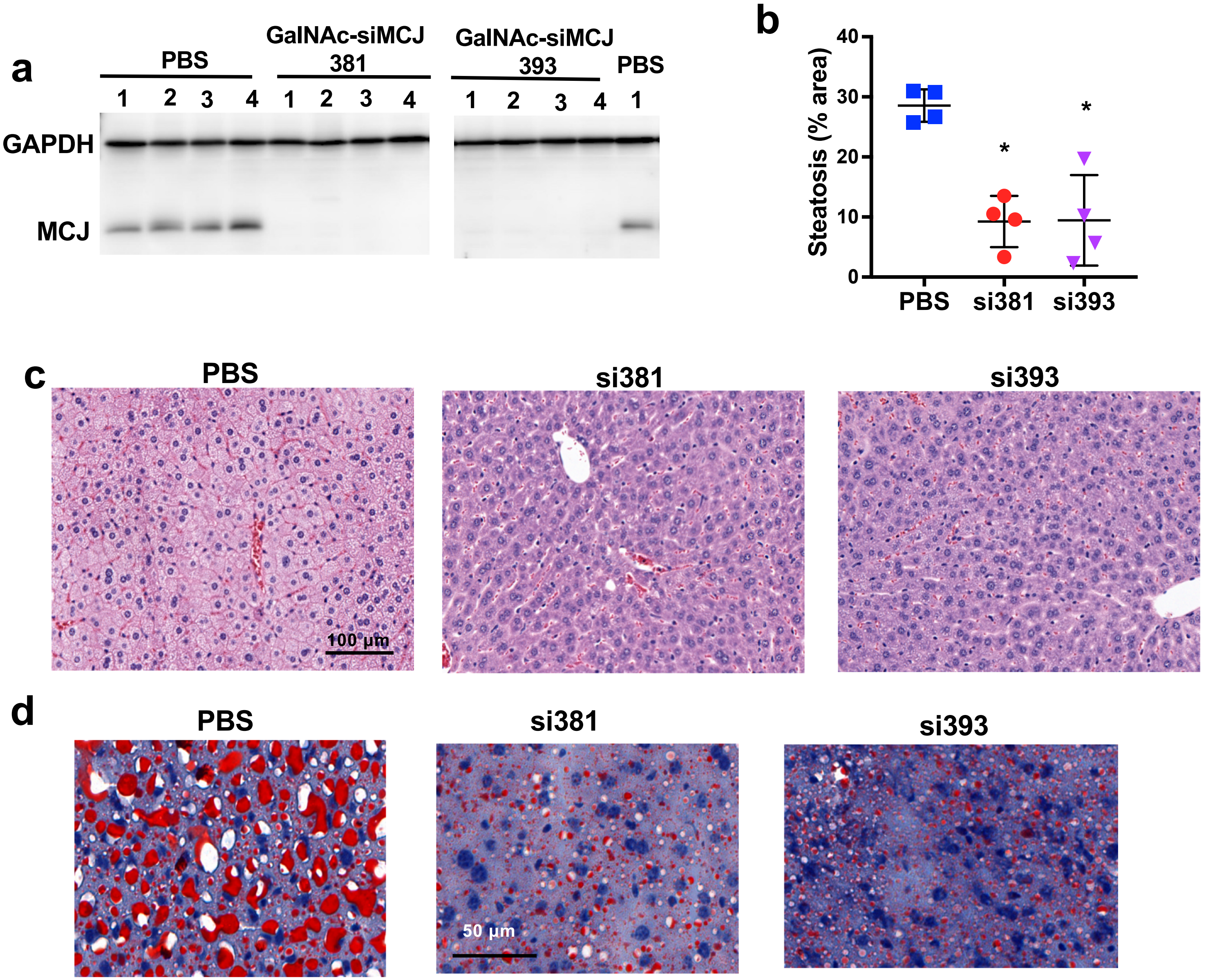

Supplementary Figure 13. Silencing MCJ expression in hepatocytes in vivo using GalNAc-formulated MCJ siRNA greatly diminishes fatty liver in the fasting model. Mice received a single s.c. dose (10 mg/Kg) of GalNAc-si381 (n=4), GalNAc-si393 (n=4), or PBS (n=4). Five days later, food was removed, and mice were harvested after 36 h of fasting. (**a**) Expression of MCJ in the livers by Western blot analysis. Each lane represents one mouse. (**b**) Representative images of H&E staining of liver sections. (**c**) Quantification of steatotic areas in the liver. (**d**) Representative images of liver stained with Oil Red O. *denotes $p<0.05$, as determined by one-way ANOVA. Error bars show standard deviation (SD).

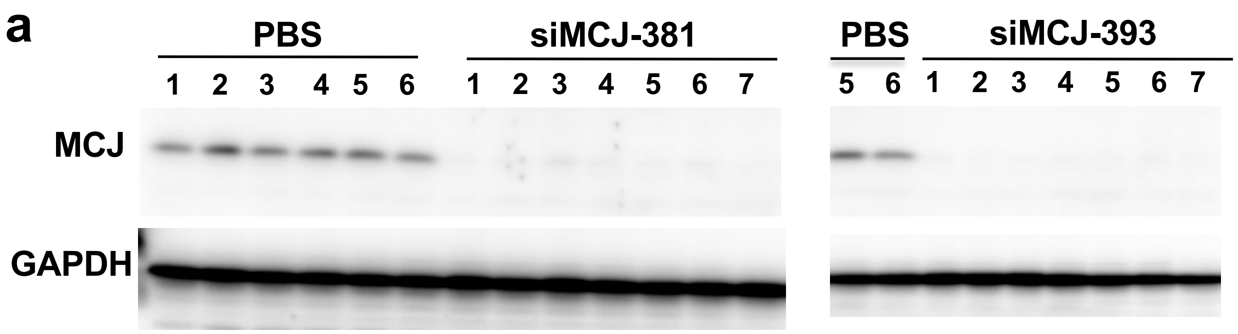

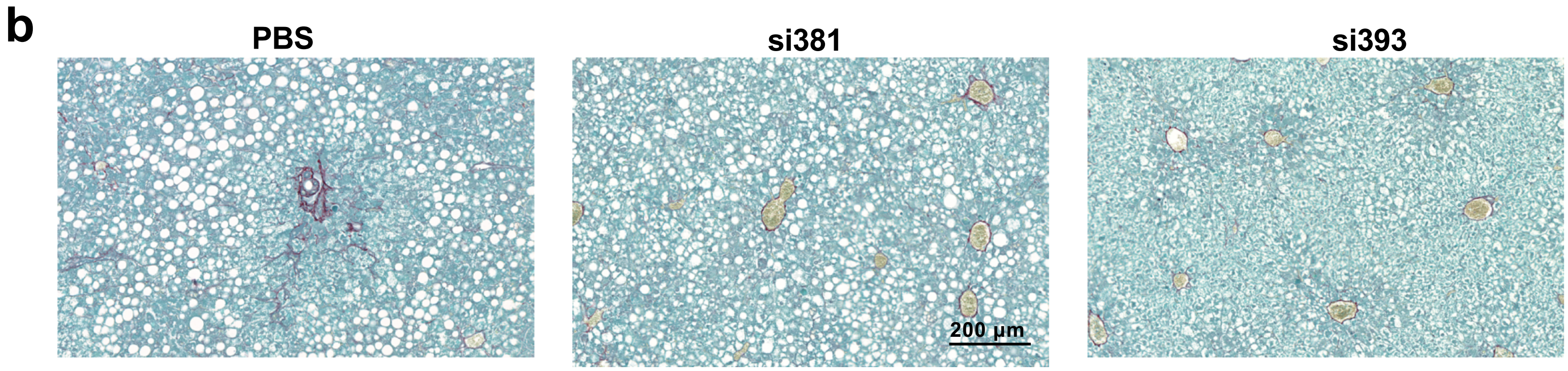

Supplementary Figure 14. Treatment with GalNAc-formulated MCJ siRNA strikingly decreases liver fibrosis development in the MCD diet model. Mice received a s.c. dose (10 mg/Kg) of GalNAc-si381 (n=7), GalNAc-si393 (n=7), or PBS (n=6) at the time they were placed on MCD diet. The mice received a second dose two weeks later and were harvested one week after the last dose. (**a**) Expression of MCJ in the livers by Western blot analysis. Each lane represents one mouse. (**b**) Representative images of liver fibrosis as determined by Picro Sirius Red staining of liver sections.

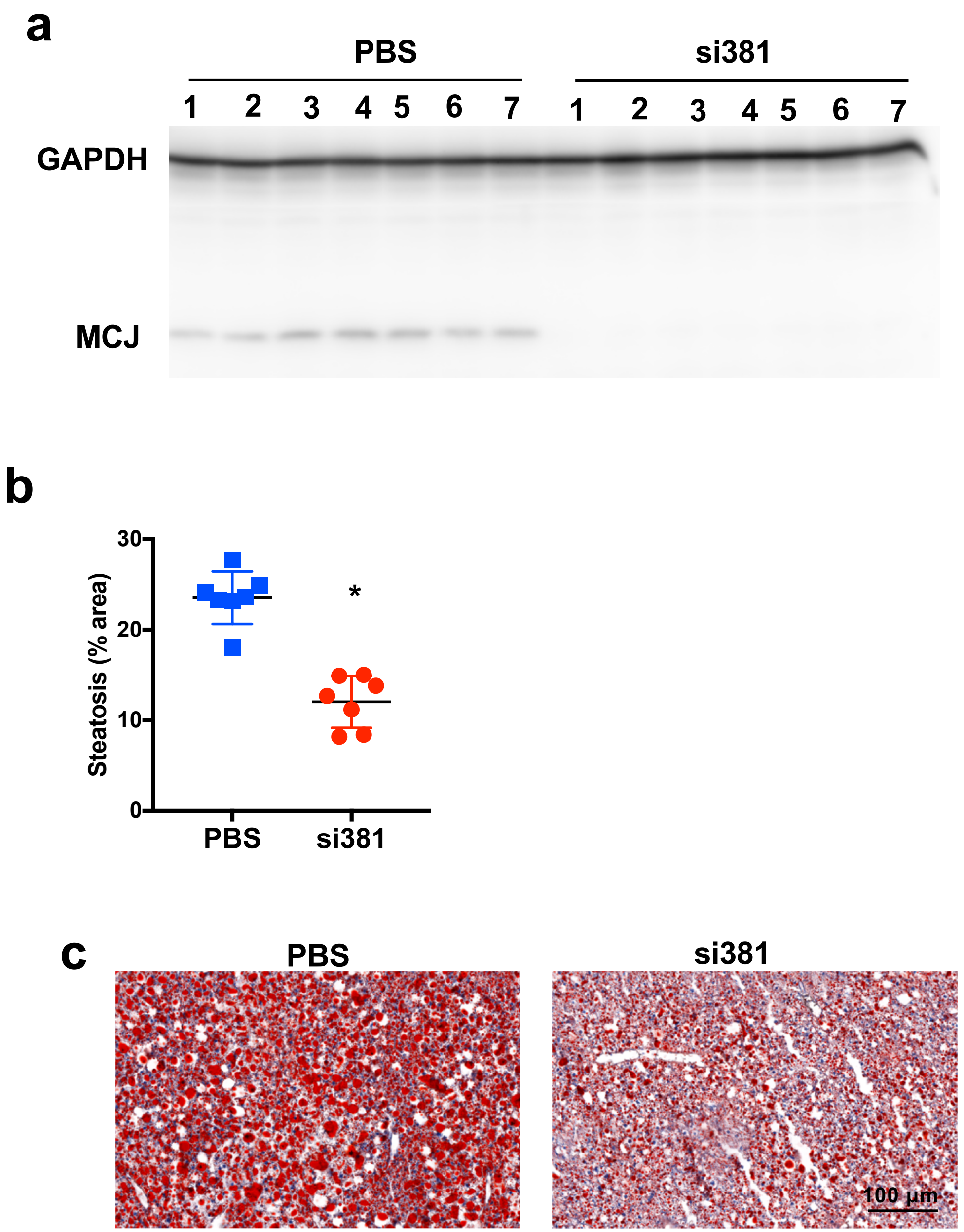

Supplementary Figure 15. Treatment with GalNAc-siMCJ decreases liver steatosis and lipid accumulation in the CD-HFD model. Mice (n=7) were placed on choline-deficient high-fat diet for 4 months prior to the treatment with GalNAc-si381 (10 mg/kg) or PBS every two weeks for 2 additional months on the diet. Tissues were harvested 2 weeks after the last dose. (**a**) MCJ levels in the livers were examined by Western blot analyses. Each lane represents one mouse. (**b**) Quantification of liver steatotic area by H&E staining, relative the total histological area. (**c**) Representative images of Oil Red O stained-liver sections. Error bars show standard deviation (SD).

Supplementary to Fig. 15

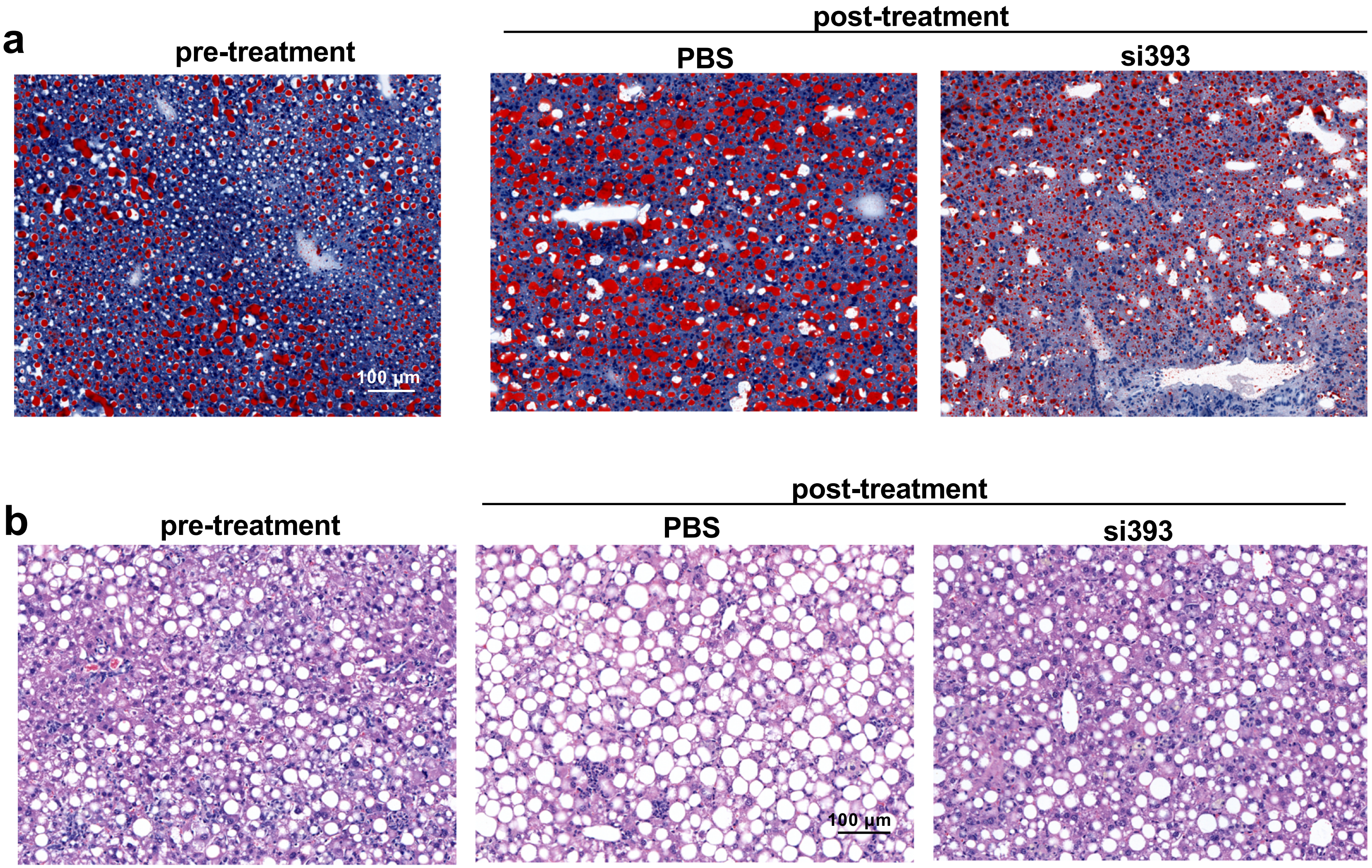

Supplementary Figure 16. Efficacy of GalNAc-formulated MCJ siRNA treatment in diminishing lipid accumulation and steatosis in the mMCD diet model. Mice (n=5) were placed on mMCD diet for 2 weeks prior to the initiation of the treatment (pre-treatment) with GalNAc-si393 (10 mg/Kg) or PBS every 2 weeks for 4 additional weeks on the diet. Tissues were harvested two weeks after the last dose (post-treatment). (**a**) Representative images of H&E stained-liver sections. (**b**) Representative images of Oil Red O stained-liver sections.

Supplementary to Fig. S16

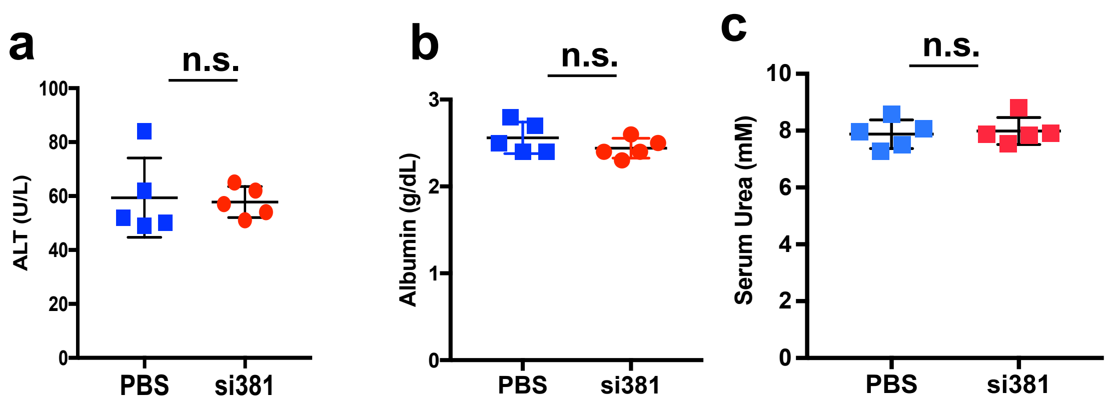

Supplementary Figure 17. Toxicity studies for GalNAc-formulated MCJ siRNA. Mice received a single s.c. high dose (30 mg/Kg) GalNAc-si393 (n=5) or PBS (n=5). (**a**) Alanine aminotransferase (ALT) levels, (**b**) albumin levels, and (**c**) urea levels in the serum were measured one week after injection. n.s. denotes not-statistically significant ($p>0.05$) as determined by Sutdent's t-test analysis. Error bars show standard deviation (SD) in all panels.

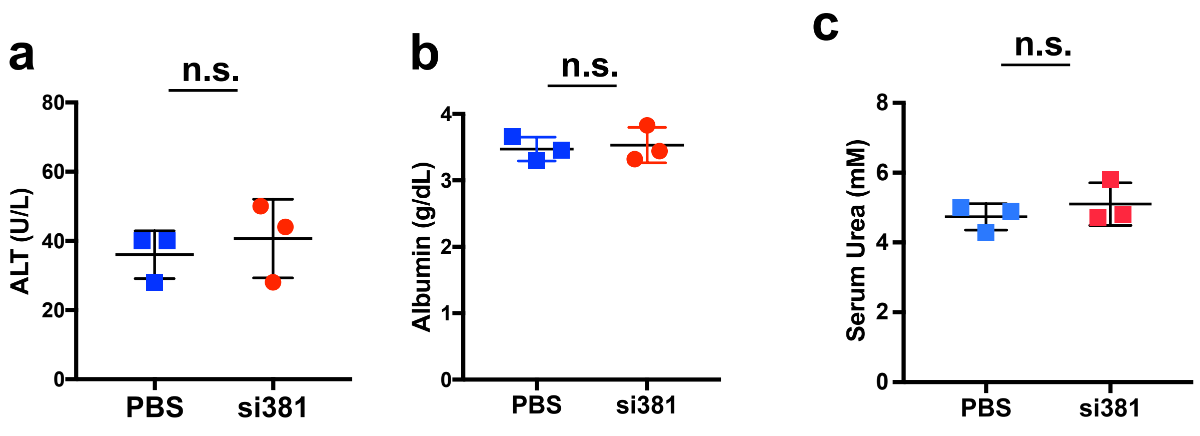

Supplementary Figure 18. Toxicity studies for GalNAc-formulated MCJ siRNA. Mice received a single s.c. high dose (30 mg/Kg) GalNAc-si393 (n=5) or PBS (n=5). (**a**) Alanine aminotransferase (ALT) levels, (**b**) albumin levels, and (**c**) urea levels in the serum were measured 24 h after injection. n.s. denotes not-statistically significant ($p>0.05$) as determined by Student's t-test analysis. Error bars show standard deviation (SD) in all panels

Supplementary Fig. 18

Supplementary Table I. List of primers used.

Gene Name	Symbol[a]		Sequence[b]
ATP Binding Cassette Subfamily D Member 1	*Abcd1*	Forward	5’-AGTGCCATCCGCTACCTAGA-3'
		Reverse	5’-CAGGGTTTCGAAGTCGTCCA-3'
Acyl-CoA Dehydrogenase Long Chain	*Acadl*	Forward	5’-GTCCGATTGCCAGCTAATGC-3'
		Reverse	5’-CACAGGCAGAAATCGCCAAC-3'
Acyl-CoA Dehydrogenase Medium Chain	*Acadm*	Forward	5’-TCAAGATCGCAATGGGTGCT-3'
		Reverse	5’-GCTCCACTAGCAGCTTTCCA-3'
Carnitine palmitoyltransferase 1A	*Cpt1*	Forward	5’-GACTCCGCTCGCTCATTCC-3'
		Reverse	5’-GAGATCGATGCCATCAGGGG-3'
Fatty Acid Transport Protein 2	*Fatp2*	Forward	5’-CCGCAGAAACCAAATGACCG-3'
		Reverse	5’-TGCCTTCAGTGGATGCGTAG-3'
Glyceraldehyde-3- phosphate dehydrogenase	*Gapdh*	Forward	5'-CGTCCCGTAGACAAAATGG-3'
		Reverse	5'-TTGATGGCAACAATCTCCAC-3'
Nuclear Factor Erythroid 2 Like 2	*Nrf2*	Forward	5’-TGTAGGGTGGGGGTACAAAG-3'
		Reverse	5’-GAATCGGCGCTAAGGAACCC-3'
PPARG Coactivator 1 Alpha	*Ppargc1a*	Forward	5’AGACAGGTGCCTTCAGTTCAC-3’
		Reverse	5’-ACCAGAGCAGCACACTCTATG-3’

[a], official gene symbol; [b], sequence of reverse and forward primers are in 5’>3’ direction.
