# Supplementary material for: Silencing hepatic MCJ attenuates non-alcoholic fatty liver disease (NAFLD) by increasing mitochondrial fatty acid oxidation
Source: Nat Commun. 2020 Jul 3;11:3360. doi: 10.1038/s41467-020-16991-2 (PMC7334216; doi:10.1038/s41467-020-16991-2)
Supplement: Supplementary file 2 — Reporting Summary [file 41467_2020_16991_MOESM2_ESM.pdf]

nature research

Corresponding author(s): Mercedes Rincon
Mari Luz Martinez-Chantal

Last updated by author(s): Feb 28, 2020

# Reporting Summary

Nature Research wishes to improve the reproducibility of the work that we publish. This form provides structure for consistency and transparency in reporting. For further information on Nature Research policies, see Authors & Referees and the Editorial Policy Checklist .

## Statistics

For all statistical analyses, confirm that the following items are present in the figure legend, table legend, main text, or Methods section.

| n/a | Confirmed | |
|---|---|---|
| ☐ | ☒ | The exact sample size (*n*) for each experimental group/condition, given as a discrete number and unit of measurement |
| ☐ | ☒ | A statement on whether measurements were taken from distinct samples or whether the same sample was measured repeatedly |
| ☐ | ☒ | The statistical test(s) used AND whether they are one- or two-sided<br>*Only common tests should be described solely by name; describe more complex techniques in the Methods section.* |
| ☒ | ☐ | A description of all covariates tested |
| ☒ | ☐ | A description of any assumptions or corrections, such as tests of normality and adjustment for multiple comparisons |
| ☐ | ☒ | A full description of the statistical parameters including central tendency (e.g. means) or other basic estimates (e.g. regression coefficient) AND variation (e.g. standard deviation) or associated estimates of uncertainty (e.g. confidence intervals) |
| ☒ | ☐ | For null hypothesis testing, the test statistic (e.g. *F*, *t*, *r*) with confidence intervals, effect sizes, degrees of freedom and *P* value noted<br>*Give P values as exact values whenever suitable.* |
| ☒ | ☐ | For Bayesian analysis, information on the choice of priors and Markov chain Monte Carlo settings |
| ☒ | ☐ | For hierarchical and complex designs, identification of the appropriate level for tests and full reporting of outcomes |
| ☐ | ☒ | Estimates of effect sizes (e.g. Cohen's *d*, Pearson's *r*), indicating how they were calculated |

*Our web collection on statistics for biologists contains articles on many of the points above.*

## Software and code

Policy information about availability of computer code

| | |
|---|---|
| Data collection | No software or code was used in data collection |
| Data analysis | GraphPad Prism version 7 was used to analyse data. The R package RCGAbiolinks, minifi, and ENmix were used to perform the methylation analysis of the MCJ gene |

For manuscripts utilizing custom algorithms or software that are central to the research but not yet described in published literature, software must be made available to editors/reviewers. We strongly encourage code deposition in a community repository (e.g. GitHub). See the Nature Research guidelines for submitting code & software for further information.

## Data

Policy information about availability of data

All manuscripts must include a data availability statement. This statement should provide the following information, where applicable:

- Accession codes, unique identifiers, or web links for publicly available datasets
- A list of figures that have associated raw data
- A description of any restrictions on data availability

No data sets with mandated depositions are present in the study. We now provide the position sequences for the Alnylam siMCJs that were conjugated to GalNac.

# Field-specific reporting

Please select the one below that is the best fit for your research. If you are not sure, read the appropriate sections before making your selection.

☒ Life sciences ☐ Behavioural & social sciences ☐ Ecological, evolutionary & environmental sciences

For a reference copy of the document with all sections, see nature.com/documents/nr-reporting-summary-flat.pdf

# Life sciences study design

All studies must disclose on these points even when the disclosure is negative.

| | |
|---|---|
| Sample size | Pilot and published studies were used to determine the sample size. The mean and variance between groups was sufficient to determine if differences between groups were significant |
| Data exclusions | In the insulin resistance studies using DIO mice obtained from Jackson Labs, one mouse in the control group was excluded because it failed to gain weight when fed with high fat diet. |
| Replication | Multiple independent experiments were conducted to verify the reproducibility of the data. All attempts at reproducibility were successful. Reproducibility of the results was also further demonstrated because about 50% of the studies in the manuscript were done in Dr. Rincon's lab at the University of Vermont, and later University of Colorado in USA, and about 50% of the studies were performed at Dr. Martinez's lab at CIC bioGUNE in Bilbao (Spain). |
| Randomization | After their initial period on a NAFLD diet, mice were assigned to control and siMCJ-treatment groups based on their weight so that each experimental group had an equivalent weight distribution. This process resulted in control and treated mice being housed in the same cage. |
| Blinding | Tissue harvest and processing were performed without referring to the treatment group. As a consequence of our randomization process, each animal cage contained both control and treated mice in an unpredictable order |

# Reporting for specific materials, systems and methods

We require information from authors about some types of materials, experimental systems and methods used in many studies. Here, indicate whether each material, system or method listed is relevant to your study. If you are not sure if a list item applies to your research, read the appropriate section before selecting a response.

## Materials & experimental systems

| n/a | Involved in the study | |
|---|---|---|
| ☐ | ☒ | Antibodies |
| ☐ | ☒ | Eukaryotic cell lines |
| ☒ | ☐ | Palaeontology |
| ☐ | ☒ | Animals and other organisms |
| ☐ | ☒ | Human research participants |
| ☐ | ☒ | Clinical data |

## Methods

| n/a | Involved in the study | |
|---|---|---|
| ☒ | ☐ | ChIP-seq |
| ☒ | ☐ | Flow cytometry |
| ☒ | ☐ | MRI-based neuroimaging |

## Antibodies

| | |
|---|---|
| Antibodies used | Anti-human MCJ, generated by the Rincon group (Hatle et al, Mol Cell Biol 2007); Anti-mouse MCJ, generated by the Rincon group (Hatle et al, Mol Cell Biol 2013); Anti-GAPDH, Santa Cruz catalog number 25778, FL-335, Lot 13015; Anti-GAPDH, Abcam catalog number ab8245; Anti-F4/80, Bio-Rad MCA497BB |
| Validation | The commercial anti-GAPDH antibodies gave a single band of the expected molecular weight and were validated by the manufacturer. The anti-human MCJ was generated by the Rincon group (Hatle et al, Mol Cell Biol 2007) and validated for western blots using human cells that either overexpressed MCJ or had reduced MCJ expression. Anti-mouse MCJ was generated by Rincon group (Hatle et al, Mol Cell Biol 2013) and validated for western blot using wild-type and MCJ-deficient mouse tissue extracts. |

## Eukaryotic cell lines

Policy information about cell lines

| | |
|---|---|
| Cell line source(s) | the study does not include cell lines |
| Authentication | N/A |
| Mycoplasma contamination | N/A |
| Commonly misidentified lines (See ICLAC register) | N/A |

# Animals and other organisms

Policy information about studies involving animals; ARRIVE guidelines recommended for reporting animal research

| | |
|---|---|
| Laboratory animals | Male C57Bl/6J mice, MCJ KO mice and Lepr/db mice between 8-24 weeks were used for these studies |
| Wild animals | No wild animals were used |
| Field-collected samples | No field-collected samples were used in the study |
| Ethics oversight | Animal procedures were approved by the University of Vermont and the University of Colorado Institutional Animal Care and Use Committee or CIC bioGUNE´s IACUC/CBBA (Órgano habilitado) and the Competent Authority (Diputación de Bizkaia) following Spanish and European regulations |

Note that full information on the approval of the study protocol must also be provided in the manuscript.

# Human research participants

Policy information about studies involving human research participants

| | |
|---|---|
| Population characteristics | Healthy human liver samples were obtained from patients with histologically healthy liver, BMI < 25 Kg/m2, normal fasting glucose, cholesterol and triglycerides, normal AST and ALT, and no evidence of viral infections (HBV, HCV and HIV). Diseased liver samples came from 21 patients with a clinical diagnosis of NAFLD who underwent a liver biopsy with diagnostic purposes. The average NAFLD patient age was 50 +/- 15 years. The patient group included 17 females and 4 males. Inclusion criteria for NAFLD patients were based on an alcohol intake lesser than 20 g/day, the presence of biopsy-proven steatosis with/without necroinflammation and/or fibrosis, and no evidence of hepatitis B, hepatitis C, or human immunodeficiency virus infection. Clinical examination included a detailed interview with special emphasis on both alcohol intake and medications use, history of known diabetes and arterial hypertension, as well as measurements of weight, height, blood pressure and waist and hip perimeters. Body mass index (BMI) was calculated as weight (Kg) divided by height (m) squared. Fasting blood samples were obtained and used to measure alanine and aspartate transaminases (ALT and AST, respectively), GGT, total cholesterol, HDL-cholesterol, triglyceride, glucose, HbA1c and insulin. Lab values are available and can be included. A table (Table I) with the patient information is now included. |
| Recruitment | Histological liver samples were obtained from patients who underwent liver biopsy for diagnostic purposes. No patient recruitment was done for these studies. |
| Ethics oversight | The Human Ethics Committee of Santa Cristina Hospital, the Comité Consultatif de Protection des Personnes dans la Recherche Biomédicale de Nice or the Human Ethics Committee of Valdecilla Hospital approved the study procedures. |

Note that full information on the approval of the study protocol must also be provided in the manuscript.

# Clinical data

Policy information about clinical studies

All manuscripts should comply with the ICMJEguidelines for publication of clinical research and a completedCONSORT checklist must be included with all submissions.

| | |
|---|---|
| Clinical trial registration | *Provide the trial registration number from ClinicalTrials.gov or an equivalent agency.* |
| Study protocol | *Note where the full trial protocol can be accessed OR if not available, explain why.* |
| Data collection | *Describe the settings and locales of data collection, noting the time periods of recruitment and data collection.* |
| Outcomes | *Describe how you pre-defined primary and secondary outcome measures and how you assessed these measures.* |
